# Differential expression of *GS5* regulates grain size in rice

**DOI:** 10.1093/jxb/erv058

**Published:** 2015-02-24

**Authors:** Chunjue Xu, Yu Liu, Yibo Li, Xiaodong Xu, Caiguo Xu, Xianghua Li, Jinghua Xiao, Qifa Zhang

**Affiliations:** National Key Laboratory of Crop Genetic Improvement and National Center of Plant Gene Research (Wuhan), Huazhong Agricultural University, Wuhan 430070, China

**Keywords:** *cis*-element, competitive interaction, differential regulation, expression variation, grain size, *GS5*.

## Abstract

Grain weight is a major determinant of grain yield. *GS5* is a positive regulator of grain size such that grain width, filling, and weight are correlated with its expression level. Previous work suggested that polymorphisms of *GS5* in the promoter region might be responsible for the variation in grain size. In this study, two single nucleotide polymorphisms (SNPs) between the wide-grain allele *GS5-1* and the narrow-grain allele *GS5-2* in the upstream region of the gene that were responsible for the differential expression in developing young panicles were identified. These two polymorphs altered the responses of the *GS5* alleles to abscisic acid (ABA) treatments, resulting in higher expression of *GS5-1* than of *GS5-2* in developing young panicles. It was also shown that SNPs in light-responsive elements of the promoter altered the response to light induction, leading to higher expression of *GS5-2* than *GS5-1* in leaves. Enhanced expression of GS5 competitively inhibits the interaction between OsBAK1-7 and OsMSBP1 by occupying the extracellular leucine-rich repeat (LRR) domain of OsBAK1-7, thus preventing OsBAK1-7 from endocytosis caused by interacting with OsMSBP1, providing an explanation for the positive association between grain size and *GS5* expression. These results advanced our understanding of the molecular mechanism by which *GS5* controls grain size.

## Highlight

Two SNPs in the promoter of *GS5* are responsible for expression variation controlling grain size. Enhanced expression of GS5 competitively inhibits the interaction between OsBAK1 and OsMSBP1, promoting grain size.

## Introduction

Grain weight is one of the most important yield traits in rice, and is determined by grain size and the degree of grain filling. Grain size is measured by grain length, width, and thickness. With the rapid advance of rice genome research, a number of quantitative trait loci (QTLs) for grain length (*GS3*) (Fan *et al.*, 2006), grain width (*GW2*, *qSW5/GW5*, *GS5*, and *GW8/OsSPL16*) (Song *et al.*, 2007; Shomura *et al.*, 2008; Weng *et al.*, 2008; Li *et al.*, 2011; Wang *et al.*, 2012), grain filling (*GIF1*) (Wang *et al.*, 2008), and grain weight (*TGW6*) (Ishimaru *et al.*, 2013) have been isolated in the past decade. Functional analyses of these genes have brought to light the molecular mechanisms by which the genes regulate grain size.

For example, *GS3*, a major negative regulator of grain length, encodes four putative domains functioning differentially in grain size regulation. It contains a plant-specific organ size regulation (OSR) domain in the N-terminus, which is both necessary and sufficient for functioning as a negative regulator. However, the tumour necrosis factor receptor/nerve growth factor receptor (TNFR/NGFR) family cysteine-rich domain and the von Willebrand factor type C (VWFC) domain in the C-terminus show an inhibitory effect on OSR function (Mao *et al.*, 2010). Two major genes negatively controlling grain width, *GW2* and *qSW5/GW5*, are likely to function in the ubiquitin–proteasome pathway, as *GW2* encodes a RING-type E3 ubiquitin ligase (Song *et al.*, 2007) and qSW5/GW5 may physically interact with polyubiquitin (Weng *et al.*, 2008). *GIF1* encodes a cell wall invertase required for carbon partitioning during early grain filling, and *TGW6* encodes an indole-3-acetic acid (IAA)-glucose hydrolase affecting the transition from the syncytial to the cellular phase of the endosperm, both of which regulate the source–sink relationship during grain filling, eventually affecting the final grain weight (Wang *et al.*, 2008; Ishimaru *et al.*, 2013). The allelic variation at the *GW8/OsSPL16* locus is a 10bp deletion in the promoter, which significantly reduces the expression level of the gene and thus the reduction in grain width (Wang *et al.*, 2012), whereas allelic variations of all other genes are caused by mutations of the structural genes that change protein sequences. Despite this progress, the details are still lacking for almost all the genes regarding the mechanistic understanding of how they regulate grain size.

Using populations derived from a cross between Zhenshan 97 and H94, Li *et al.* (2011) cloned a minor QTL, *GS5*, on the short arm of chromosome 5 for grain width, filling, and weight. The grains of the near-isogenic line NIL(ZS97) are 8.7% wider and 7.0% heavier than those of NIL(H94), and the grain-filling rate is significantly higher in NIL(ZS97), leading to a 7.4% increase in grain yield per plant (Li *et al.*, 2011).

*GS5* encodes a putative serine carboxypeptidase-like (SCPL) protein, a member of a large family characterized by a conserved serine–aspartate–histidine catalytic triad (Fraser *et al.*, 2005; Feng and Xue, 2006; Tripathi and Sowdhamini, 2006). The alleles from both Zhenshan 97 (wide grain) and H94 (narrow grain) are predicted to be full length, and overexpression of either allele could increase grain width (Li *et al.*, 2011). Based on this result, it was concluded that *GS5* is a positive regulator of grain size, and a higher expression level is correlated with increased grain width.

The study reported herein was attempted in order to investigate the functional relationship between grain size and the transcription level of *GS5*. Two single nucleotide polymorphisms (SNPs) located in the flanking sequence of a putative gibberellin- (GA) responsive element in the promoter were identified that altered the response of the *GS5* alleles to abscisic acid (ABA) suppression, causing differential expression of the *GS5* alleles in young panicles. Subcellular localization and protein–protein interaction assay indicated that enhanced expression of GS5 competitively inhibits the interaction between OsBAK1-7 and OsMSBP1 by occupying the extracellular leucine-rich repeat (LRR) domain of OsBAK1-7, thus preventing OsBAK1-7 from endocytosis caused by interacting with OsMSBP1. These results provided an explanation for the positive association between grain size and the level of *GS5* expression.

## Materials and methods

### Field planting and trait measurement

Rice plants were grown and examined under natural field conditions in the experimental station of Huazhong Agriculture University, Wuhan, China. The planting density was 16.5cm between plants in a row and the rows were 26cm apart. Harvested grains were air-dried and stored at room temperature before testing. Thirty randomly chosen, fully filled grains from each plant were used for grain size measurement. Every 10 grains were lined up length-wise along a vernier caliper to measure grain length and then arranged by breadth to measure grain width.

### Constructs and transformation

The coding sequences (CDS) of *GS5* from H94 and Zhenshan 97, and that of *OsBAK1-7* and *OsMSBP1* from Zhenshan 97 were amplified by reverse transcription–PCR (RT–PCR). Both *GS5* sequences were inserted into a modified plant binary vector pU1301 (Sun and Zhou, 2008) that contains a maize ubiquitin gene promoter and a 3× FLAG-tag located downstream in-frame to construct the *P*_*Ubi*_ *::GS5-FLAG* vectors. The *GS5* and *OsBAK1-7* CDSs were fused in-frame at their C-terminus with green fluorescent protein (GFP) under the control of the *Cauliflower mosaic virus* (CaMV) 35S promoter in another modified plant binary vector pDX2181 (Ye *et al.*, 2012) to construct the *P*_*35S*_ *::GS5-GFP* and *P*_*35S*_ *::OsBAK1-7-GFP* vectors. For co-expression of OsBAK1-7 and OsMSBP1, the *OsMSBP1* CDS was fused with red fluorescent protein (RFP) at its C-terminus and inserted downstream of a CaMV35S promoter; the *P*_*35S*_ *::OsMSBP1-RFP* fragment was then cloned into the *P*_*35S*_ *::OsBAK1-7-GFP* vector. For promoter strength analysis, the truncated *GS5* promoter fragments were amplified from H94 and Zhenshan 97 and fused to β-glucuronidase (GUS) in pDX2181. The fraction-replaced promoter fragments were generated by restriction enzyme digestion and ligation at *Hin*dIII (–2002bp), *Ban*II (–1139bp), *Eco*T22I (–931bp), *Xba*I (–604bp), and *Bam*HI (–1bp) sites based on the constructs HH and ZZ, which carried the 2kb promoter fragment of H94 and Zhenshan 97, respectively. The mutated promoter fragments were generated by PCR site-directed mutagenesis. All these constructs were confirmed by sequencing, introduced into the *Agrobacterium tumefaciens* strain EHA105 by electroporation, and then introduced into Zhonghua 11 by *Agrobacterium*-mediated transformation as described in Lin and Zhang (2005) with minor modifications, or infiltrated into *Nicotiana tabacum* epidermal cells as previously described (Sparkes *et al.*, 2006).

### Transient expression in tobacco BY-2 protoplasts

The *GS5* CDSs of H94 and Zhenshan 97 were cloned into the pM999-EGFP vector under the control of the CaMV35S promoter, fused in-frame at their C-terminus with GFP. The closed circular plasmid DNA was purified by equilibrium centrifugation in CsCl–ethidium bromide gradients as described in Sambrook *et al.* (1989). Tobacco BY-2 protoplast generation and purification, transformation of plasmid DNA into protoplasts via electroporation, and incubation of protoplasts for protein analysis were carried out as previously described (Miao and Jiang, 2007).

### Gene expression analysis

For expression analysis, fresh tissues of NIL(H94) and NIL(ZS97) were harvested at 17:00h to 19:00h and stored at –70 °C before testing. For light conditions and plant hormone treatments, seeds of NIL(H94) and NIL(ZS97) were soaked in water at 30 °C for 2 d, and grown hydroponically to the trefoil stage at 26 °C. For diurnal expression analysis, half of the plants were incubated under long-day conditions (14h light/10h darkness) in an illumination incubator and the other half were incubated under short-day conditions (10h light/14h darkness) in another illumination incubator. For the hormone treatments, seedlings were incubated under long-day conditions and 10 μM GA_3_, ABA, or brassinosteroid (BR) was added to the nutrient solution with ddH_2_O as negative control. The third leaf blades were harvested from three different plants for each treatment at the specified time points and stored in liquid nitrogen. RNA isolation, reverse transcription, and quantitative real-time PCR were carried out as previously described (Mao *et al.*, 2010). GS5qF and GS5qR were used to amplify the transcript of *GS5*, and Act1F and Act1R for *Actin1* as the internal control.

### Protein expression analysis

Expression of GS5-FLAG or GS5–GFP in young panicles of transgenic plants or tobacco leaves was analysed using monoclonal anti-FLAG M2 primary antibody (Sigma-Aldrich) and goat anti-mouse IgG secondary antibody (SouthernBiotech) or anti-GFP primary antibody (Abcam) and goat anti-rabbit IgG secondary antibody (Southern Biotech) following Huang *et al.* (2007). Fluorescence signals in rice lemma or tobacco leaf epidermal cells and BY-2 protoplasts were observed using a Leica TCS SP2 AOBS confocal microscope (Leica Microsystems) according to the user manual. Plasmolysis was induced by the addition of 1M mannitol solution to the tobacco leaf lower epidermis slice. For quantitative analysis of GUS activity, young panicles of 20cm in length from transgenic plants were harvested; total protein was extracted and quantified as described in Ye *et al.* (2012) and used for fluorometric assay according to the method described by Jefferson *et al.* (1987) with an Infinite 200 photometer (Tecan).

### Yeast two-hybrid assay

Total RNA from young panicles of 2cm in length from Zhenshan 97 was isolated for generation of a cDNA library with BD Matchmaker™ Library Construction & Screening Kits (Clontech). The putative A-chain CDS of *GS5* was cloned into the pGBKT7 vector and tested for transcriptional activation and toxicity as described in the user manual. Screening for two-hybrid interactions was carried out by yeast mating, and positive interactions were verified and analysed by series strategies according to the user manual of the kit. The putative B-chain CDS of *GS5*, the LRR domain CDS, and the kinase domain CDS of *BAK1* homologues were cloned into pGADT7 and co-transformed with pGBKT7 A-chain to retest the interactions in yeast.

## Results

### Differential expression pattern of *GS5*

**Fig. 1.**
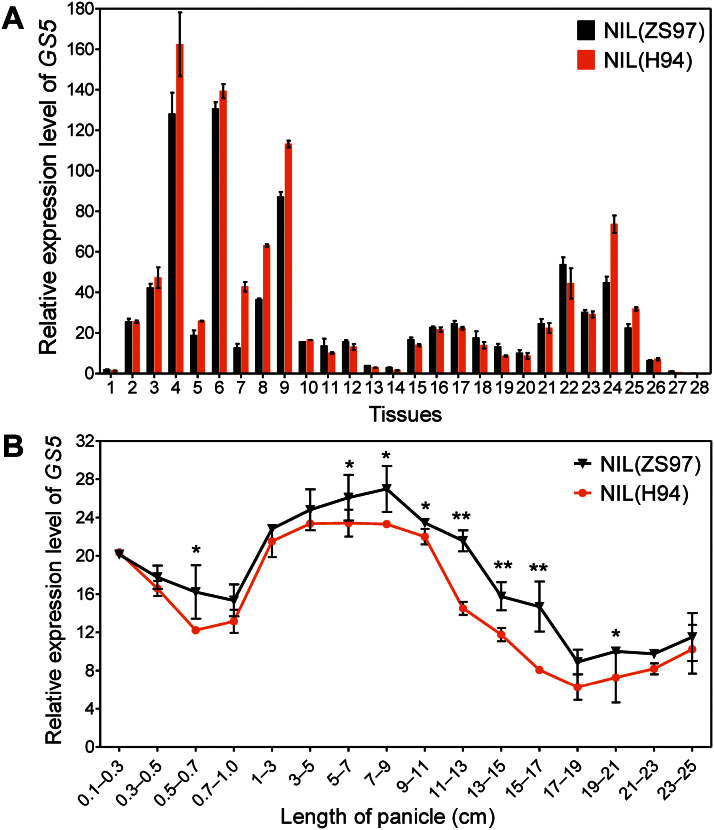
Expression pattern of the two *GS5* alleles. Fresh tissues of NIL(ZS97) and NIL(H94) grown under natural field conditions were used. (A) Expression pattern of *GS5* during the entire life cycle of the rice plant. 1, Embryo at 72h after imbibition; 2, plumule at 48h after emergence; 3, leaf from seedlings at the single-leaf stage; 4, leaf blade at the trefoil stage; 5, young leaf blade at the tillering stage; 6, mature leaf blade at the tillering stage; 7, flag leaf blade at the heading stage; 8, leaf sheath at the trefoil stage; 9, leaf sheath at the tillering stage; 10, tiller bud at the tillering stage; 11, radicle at 48h after emergence; 12, root at the trefoil stage; 13, young culm at the booting stage; 14, young culm at the heading stage; 15, developing panicle of 0.1–1cm in length; 16, developing panicle of 1–5cm in length; 17, developing panicle of 5–10cm in length; 18, developing panicle of 10–15cm in length; 19, developing panicle of 15–20cm in length; 20, developing panicle of 20–25cm in length; 21, panicle at the heading stage; 22, panicle on the day of flowering; 23, hull at 2 days after pollination (DAP); 24, hull at 5 DAP; 25, hull at 8 DAP; 26, endosperm at 2 DAP; 27, endosperm at 5 DAP; 28, endosperm at 8 DAP. (B) Comparison of *GS5* transcripts in NIL(ZS97) and NIL(H94) during young panicle development. All data are presented as the mean ±SE (*n* ≥3). *P<0.05; **P<0.01, *t*-test. (This figure is available in colour at *JXB* online.)

Li *et al.* (2011) showed that *GS5*-controlled grain size variation is positively correlated with its expression level. To understand how the difference in expression levels between the *GS5* alleles is related to grain size, the temporal and spatial expression patterns of *GS5* were assayed and it was found that in general it had a much higher expression level in green tissues than in non-green tissues, such as culm, root, young panicle, and endosperm (Fig. 1A). In non-green tissues, the highest expression of *GS5* was detected in young panicles of 1–11cm in length (Fig. 1A), during which increase the volume of the lemmas/paleae increased rapidly. After that the *GS5* transcript decreased gradually and reached the lowest level in 20cm long panicles. The lemmas/paleae reached almost their final size in the 20cm long panicle, and gradually turned green afterwards. The expression of *GS5* in the green lemmas/paleae was remarkably up-regulated at the heading stage (Fig. 1A).

Throughout development of the young panicle, the *GS5* transcript in the developing panicle was more abundant in NIL(ZS97) than in NIL(H94) (Fig. 1B), consistent with the fact that NIL(ZS97) produced wider grains. Interestingly, in leaves, the *GS5* transcript had a lower level in NIL(ZS97) (Fig. 1A), implying that the expression of *GS5* was differentially regulated in green leaves and developing panicles by different regulatory elements.

### Induction of *GS5* expression by light

**Fig. 2.**
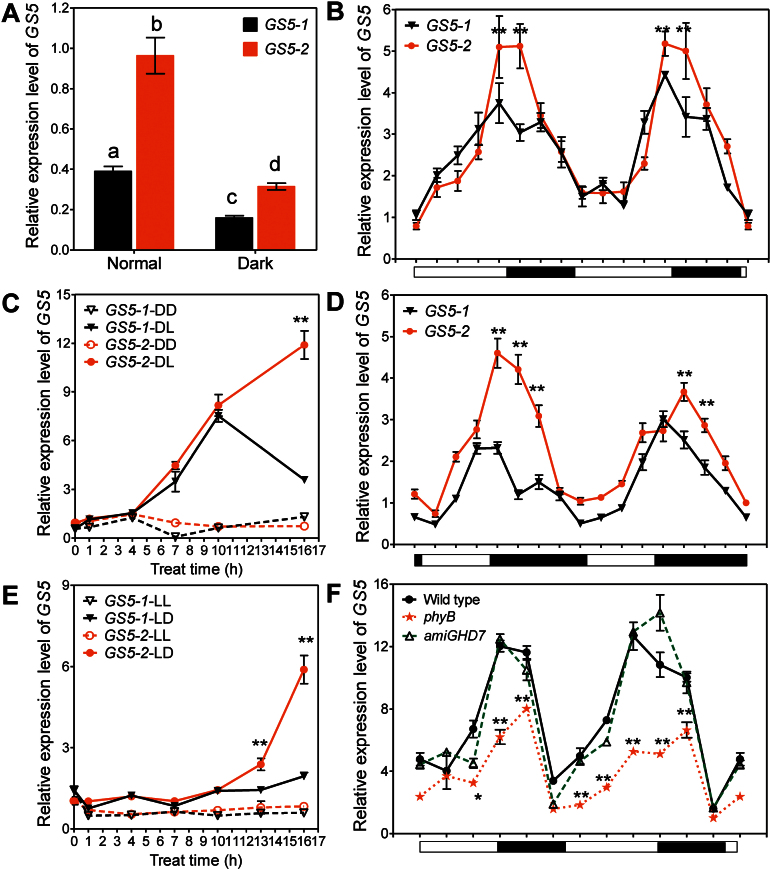
Light induction of *GS5* expression in the leaf. Fifteen-day-old seedlings of NIL(H94) and NIL(ZS97) grown under different light conditions were used for the various light treatments presented. (A) Expression of *GS5* in seedlings cultivated under long days (14h light/10h darkness) (Normal) and continuous darkness (Dark). (B, D) Diurnal expression patterns of *GS5* in leaves under long-day (B) and short-day (10h light/14h darkness) (D) conditions. (C, E) Induced expression of *GS5* in leaves by light. (C) DD, seedlings were grown in continuous darkness; DL, after 15 d of continuous darkness, seedlings were shifted to continuous illumination at time point 0. (E) LL, seedlings were grown under continuous illumination; LD, after 15 d of continuous illumination, seedlings were shifted to continuous darkness at time point 0. (F) Suppression of *GS5* transcript in a *phyB* mutant. All data are presented as the mean ±SE (*n* ≥3). *P<0.05; **P<0.01; different letters above the bars, *P*<0.01, *t*-test. (This figure is available in colour at *JXB* online.)

In order to determine the regulatory elements of *GS5* expression, the promoter region (~2kb fragment upstream of the translation start site) was analysed using PlantCARE (Lescot *et al.*, 2002). Many light-responsive elements were identified, and the polymorphisms between the two *GS5* alleles caused different numbers of light-responsive elements (Supplementary Table S1 available at *JXB* online). Seedlings cultivated in the dark had significantly less *GS5* transcript than those cultivated under normal light conditions (Fig. 2A), suggesting that light may contribute to the higher expression level in green tissues. For ease of description, the *GS5* allele from Zhenshan 97 was designated *GS5-1* and that from H94 was designated *GS5-2*.

The diurnal expression pattern of *GS5* was analyzed during a 24h period in leaves. The *GS5* transcript increased gradually in the daytime, reached its peak value at dusk, and then declined gradually until it fell to the minimum at dawn (Fig. 2B). Both *GS5* alleles had circadian rhythms, though *GS5-2* had a higher peak value. The length of the photoperiod had no impact on the circadian rhythms of *GS5* (Fig. 2B, D). The circadian rhythm disappeared when the seedlings were cultivated under continuous darkness or illumination, under which conditions *GS5* expression only stayed at its basal level (Fig. 2C, E). Changing the light conditions could induce expression of *GS5* in several hours, either from continuous darkness to illumination or from continuous illumination to darkness; the up-regulation of *GS5-2* expression was more dramatic than that of *GS5-1* (Fig. 2C, E). These results were consistent with the fact that *GS5-2* had more light-responsive elements, and thus may be more sensitive to light induction. It was also found that the *GS5* transcript in leaves was significantly suppressed in a *phyB* mutant, but not influenced in a *GHD7*-silenced transgenic plant (Weng *et al.*, 2014) (Fig. 2F), suggesting that *GS5* might function downstream in the *PHYB* pathway.

Although *GS5* positively regulated mitosis in the panicle (Li *et al.*, 2011), the differential expression in leaves between the NILs did not affect leaf size (Table 1), implying that *GS5* may have a different cellular function in leaves. Interestingly, the *phyB* mutant exhibited increased grain size (both grain width and length), and high grain chalkiness with reduced plumpness (Supplementary Fig. S1 at *JXB* online), suggesting that the *PHYB* pathway somehow influenced both grain size and filling.

**Table 1.** Grain and leaf size of transgenic positive and negative plants in the T_1_ generation

Genotype	No. of plants	10-Grain width (mm)	10-Grain length (mm)	Flag leaf length (cm)	Flag leaf width (mm)
*P*_*Ubi*_ *::GS5-1-FLAG* (+)	45	35.43±0.07	74.95±0.36	26.44±0.40	10.57±0.08
*P*_*Ubi*_ *::GS5-1-FLAG* (–)	16	33.60±0.03	73.52±0.65	26.06±0.37	11.18±0.10
*P*-value		7.75E-33	0.06	0.82	0.20
*P*_*Ubi*_ *::GS5-2-FLAG* (+)	43	35.30±0.06	75.28±0.43	26.06±0.41	10.90±0.14
*P*_*Ubi*_ *::GS5-2-FLAG* (–)	22	33.71±0.05	74.71±0.31	26.57±0.43	11.16±0.12
*P*-value		4.44E-30	0.46	0.38	0.89
*P*_*35S*_ *::GS5-1-GFP* (+)	44	35.29±0.22	76.82±0.65	–	–
*P*_*35S*_ *::GS5-1-GFP* (–)	18	33.64±0.16	75.31±0.39	–	–
*P*-value		3.42E-10	0.06		
*P*_*35S*_ *::GS5-2-GFP* (+)	48	35.37±0.19	75.28±0.41	–	–
*P*_*35S*_ *::GS5-2-GFP* (–)	22	33.53±0.12	75.62±0.63	–	–
*P*-value		3.00E-11	0.73		

(+) and (–) represent transgenic positive and negative plants, respectively.

The average values with standard error are shown (*n* ≥3).

The *P*-values are derived from Student’s *t*-tests between transgenic positive and negative plants.

### Key nucleotide polymorphisms responsible for differential expression of *GS5* in young panicles

**Fig. 3.**
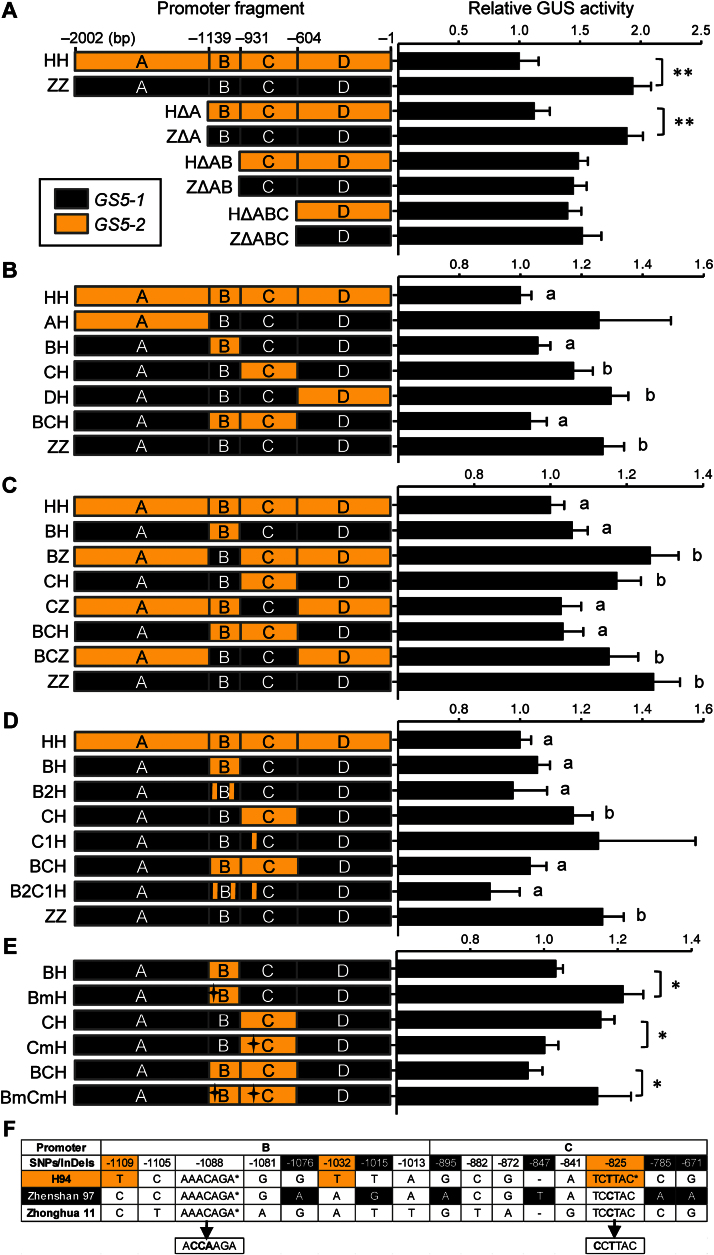
Activity of the truncated and chimeric promoter fragments of *GS5*. (A–E) Relative GUS activity of the truncated and chimeric promoter fragments of *GS5*. Constructs HH and ZZ carrying the 2kb promoter fragments from H94 and Zhenshan 97, respectively, were used as references. (A) The 2kb promoter was divided into four fractions, A (–2002bp to –1139bp), B (–1139bp to –931bp), C (–931bp to –604bp,) and D (–604bp to –1bp), and constructs with 5′ deletions were prepared accordingly. (B) The fractions A, B, C, and D of ZZ were replaced with the corresponding sequences from HH to construct AH, BH, CH, and DH, respectively. In construct BCH, fractions B and C of ZZ were replaced with the corresponding sequences from HH. (C) Either or both fractions B and C of HH were replaced with the corresponding sequences from ZZ to construct BZ, CZ, and BCZ. (D) SNP_–1109 and SNP_–1032 in fraction B of constructs B2H and B2C1H and SNP_–825 in fraction C of constructs C1H and B2C1H were changed from ZZ to the *GS5-2* genotype. (E) The putative GA-responsive element in fraction B of BH and BCH and the light-responsive element in fraction C of CH and BCH were mutated to constructs BmH, CmH, and BmCmH. The mutated *cis*-acting elements are indicated by an asterisk. All data are presented as the mean ±SE (*n* ≥15). *P<0.05; **P<0.01; different letters on the bars, *P* <0.05, *t*-test. (F) Polymorphisms in fractions B and C among H94, Zhenshan 97, and Zhonghua 11. The specific polymorphisms of H94 and Zhenshan 97 are highlighted in grey and black, respectively. The putative GA-responsive element (–1088, AAACAGA) and light-responsive element (–825, TCTTAC) are indicated by asterisks and the mutated forms are shown at the bottom. (This figure is available in colour at *JXB* online.)

To investigate the nucleotides responsible for up-regulation of *GS5-1* in the young panicle, the 2kb promoter was divided into four fractions (A, B, C, and D) (Fig. 3A), to construct serial 5′ deletions fused with the GUS reporter gene, which were introduced into Zhonghua 11, an *Oryza sativa* L. ssp. *japonica* variety suitable for transformation. Young panicles of 20cm in length from transgenic plants were harvested and used for GUS activity assay. The results showed that the expression difference between the two alleles was retained in deletions up to –1139bp, but was lost at –931bp (Fig. 3A), indicating that polymorphisms in fraction B must be responsible for the higher expression of *GS5-1*.

To validate these results, the four fractions of *GS5-1* were replaced with the corresponding sequences from *GS5-2*, and it was found that the replacements of fraction B reduced the *GS5-1* promoter strength significantly (Fig. 3B). Conversely, replacement of fraction B of *GS5-2* with the corresponding sequences from *GS5-1* raised the *GS5-2* promoter strength (Fig. 3C). As most of the light-responsive elements were located in fraction C, which must be responsible for the light-related expression in leaves, the impact of fraction C on the promoter strength in panicles was also tested. The results showed no significant change when fraction C was replaced with the counterpart from the other genotype (Fig. 3B, C); thus the promoter strength in the panicle was only related to the sequence of fraction B.

There were four SNPs in fraction B between *GS5-1* and *GS5-2* (Fig. 3F). To narrow down the range of candidate sites, the 2kb promoter of *GS5-1* and *GS5-2* was compared with the *GS5* allele from Zhonghua 11, *GS5-3*, another wide grain variety (Li *et al.*, 2011). Seven polymorphisms occurred between *GS5-2* and the other two varieties; two of them (SNP_–1109 and SNP_–1032) were in fraction B and one (SNP_–825) in fraction C (Fig. 3F). These three sites of *GS5-1* were mutated into the *GS5-2* genotype (Fig. 3D). The results showed that the mutated *GS5-1* promoter strength was reduced in the same way as replacements of the entire fraction (Fig. 3D), indicating that SNPs at –1109 and –1032 in fraction B were the key nucleotides responsible for differential expression of *GS5-1* and *GS5-2* in developing young panicles.

### Suppression of *GS5* expression by plant hormones

SNP_–1109 and SNP_–1032 in fraction B were located in the flanking sequence of a putative GA-responsive element (Fig. 3F). To examine how these two sites in fraction B affect the expression of *GS5*, the putative GA-responsive element in construct BH was mutated (Fig. 3E, F). Compared with construct BH, construct BmH with a mutated GA-responsive element resulted in higher GUS activity, implying that the putative GA-responsive element might function as a transcription repressor (Fig. 3E). Interestingly, SNP_–825 in fraction C resulted in a change of the light-responsive element, thus this *cis*-acting element was missing in *GS5-1* (Fig. 3F). This light-responsive element was also mutated in construct CH, which carried fraction C from *GS5-2* in the *GS5-1* promoter backbone, and it was found that construct CmH with the element mutated had lower GUS activity than construct CH (Fig. 3E, F), indicating that the light-responsive element promoted activation of transcription, which was in agreement with the previous results. When both *cis-*acting elements were mutated in construct BmCmH, increased GUS activity was observed, suggesting that the putative GA-responsive element was the limiting factor for *GS5* transcription in young panicles (Fig. 3E).

**Fig. 4.**
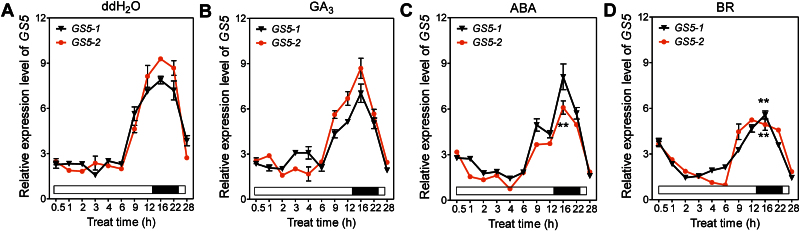
Expression level of *GS5* in response to plant hormone treatments. Fifteen-day-old seedlings of NIL(ZS97) and NIL(H94) grown under long-day conditions were treated with ddH_2_O (A), 10 μM GA_3_ (B), 10 μM ABA (C), or 10 μM BR (D). All data are presented as the mean ±SE (*n* ≥3). *P<0.05; **P<0.01, *t*-test. (This figure is available in colour at *JXB* online.)

To determine the role of the putative GA-responsive element, the expression of *GS5* was examined after plant hormone treatments. The results showed that both *GS5-1* and *GS5-2* failed to respond to GA (Fig. 4A, B). However, the expression of *GS5-2*, but not *GS5-1*, was obviously suppressed by ABA, resulting in a lower transcript level than *GS5-1* (Fig. 4A, C). Published results had proved that transcription activated by GA could be inhibited by ABA via a GA-responsive element (Jacobsen and Beach, 1985; Skriver *et al.*, 1991; Gubler and Jacobsen, 1992; Washio and Ishikawa, 1994). Therefore, it was concluded that SNP_–1109 and SNP_–1032 in fraction B altered the response of *GS5* to ABA.

It was also detected that BR suppressed the expression of both *GS5-1* and *GS5-2* (Fig. 4A, D). It was previously demonstrated that BZR1 and BES1/BZR2 were the key transcription factors for BR-regulated gene expression (He *et al.*, 2005; Yin *et al.*, 2005). It was found that BZR1 binds to the BR response element (BRRE; CGTGT/CG) (He *et al.*, 2005) and BES1/BZR2 binds to the E-box (CANNTG) (Yin *et al.*, 2005). The BRREs were usually present in BR-repressed genes and the E-boxes were present in both BR-repressed and BR-induced genes (Yu *et al.*, 2011). Eight E-boxes were found in fractions A and B in the promoter region of *GS5* (Supplementary Table S1 at *JXB* online), suggesting that *GS5* was probably one of the target genes of the BR signalling pathway.

### Secretion of GS5–GFP to the cell surface

Epitope tags were used for visualization of the GS5 proteins. To ensure that addition of epitope tags would not affect the function of the GS5 protein, constructs for both GS5-1 and GS5-2 fused to FLAG-tag driven by the ubiquitin promoter were generated, and they were expressed in Zhonghua 11. The grain width of transgenic positive plants of both constructs significantly increased, and also co-segregated with the higher expression level of *GS5* in the T_1_ progeny (Table 1), just like the original *GS5* CDSs (Li *et al.*, 2011). An increase in grain width in transgenic plants harbouring *P*_*35S*_ *::GS5-1-GFP* or *P*_*35S*_ *::GS5-2-GFP* was also observed (Table 1). Thus, higher expression of both epitope tag constructs produced wider grains just like the GS5 proteins, indicating that the GS5–epitope tag fusion proteins were functionally equivalent to GS5 *in planta*.

**Fig. 5.**
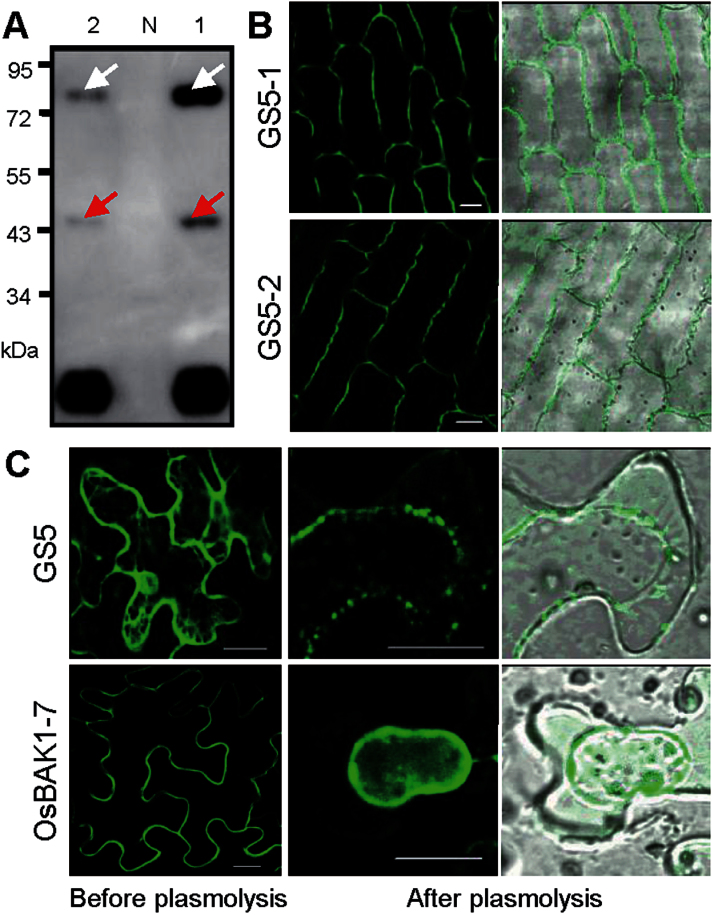
Subcellular localization of the GS5 protein. (A) Western blot assay of GS5–GFP transgenic plants using anti-GFP antibody showing the cleavage GS5–GFP into two chains (A and B) as in many SCPL-II proteins. 1, *P*_*35S*_ *::GS5-1-GFP*; N, negative control; 2, *P*_*35S*_ *::GS5-2-GFP*; the white arrow indicates the full-length GS5–GFP protein; the red arrow indicates the C-terminus of GS5 with the B-chain–GFP peptide. (B) Localization of GS5-1–GFP and GS5-2–GFP on the spikelet epidermal cell surface of transgenic plants. Scales bars=20 μm. (C) Localization of transiently expressed GS5–GFP in tobacco leaf epidermal cells showing the distribution both on the cell surface and in the internal space, whereas OsBAK1-7–GFP is found only on the cell surface (left panel). After induction of plasmolysis, the GS5–GFP signal is mostly found in the plasma membrane and a portion of the signal is detected outside the plasma membrane, whereas OsBAK1-7–GFP is found only on the plasma membrane (right panel). Scales bars=20 μm.

Phylogenetic analysis suggested that GS5 belonged to SCPL group II proteins, the same group as *Arabidopsis* BRS1 (Supplementary Fig. S2 at *JXB* online), a secreted and active serine carboxypeptidase which plays a role in the BR signalling pathway (Li *et al.*, 2001; Zhou and Li, 2005). Using an anti-GFP antibody to analyse the total protein extracts from transgenic plants, two specific bands were detected, indicating that GS5 could be cleaved into two chains (A and B) after a predicted processing step as in many SCPL-II proteins such as BRS1 (Zhou and Li, 2005). The 80kDa band represented the intact GS5–GFP protein and the 47kDa band represented the B-chain, the C-terminal fragment of GS5 attached to the GFP-tag after processing (Fig. 5A), while the A-chain could not be detected. A similar result was also obtained in a western blot assay of GS5-FLAG transgenic plants using anti-FLAG antibody (Supplementary Fig. S3A). These results indicated that the GS5–GFP fusion protein had functional integrity and the subcellular localization of GS5 could be viewed using GS5–GFP.

To investigate the subcellular localization of GS5, lemmas from young panicles of 15cm in length from GS5–GFP transgenic plants were used for confocal microscopy assay. Green fluorescence signals of GS5-1–GFP and GS5-2–GFP were detected on the cell surface (Fig. 5B). Since it was difficult to induce plasmolysis in rice lemma epidermal cells, *P*_*35S*_ *::GS5-GFP* was transiently expressed in tobacco leaf epidermal cells, using *P*_*35S*_ *::OsBAK1-7-GFP*, a homologue of plasma membrane protein bri1-associated receptor kinase 1 (BAK1) as a reference (Li *et al.*, 2002; Nam and Li, 2002). The majority of green fluorescence signal of GS5–GFP was localized on the cell surface, and there was also an obvious net-like signal inside the cell (Fig. 5C, left panel). After induction of plasmolysis, the OsBAK1-7–GFP signal moved with the plasma membrane (Fig. 5C, right panel), indicating that OsBAK1-7–GFP was localized in the plasma membrane, whereas the GS5–GFP signal was mostly found aggregating in the plasma membrane and a portion of the signal was detected outside the plasma membrane (Fig. 5C, right panel).

To demonstrate whether the net-like signal was endoplasmic reticulum (ER), the GS5–GFPs were co-expressed with the ER marker RFP–HDEL (Supplementary Fig. S4A at *JXB* online) or the *cis-*Golgi marker Man1–RFP (Supplementary Fig. S4B) in tobacco BY-2 protoplasts. The results confirmed that GS5–GFPs were co-located with RFP–HDEL (Supplementary Fig. S4A). These observations indicated that GS5 was secreted from the ER to the cell surface, and the plasma membrane signal might result from its interaction with some plasma membrane protein(s).

### Interaction between GS5 and the extracellular domain of BAK1 homologues

**Fig. 6.**
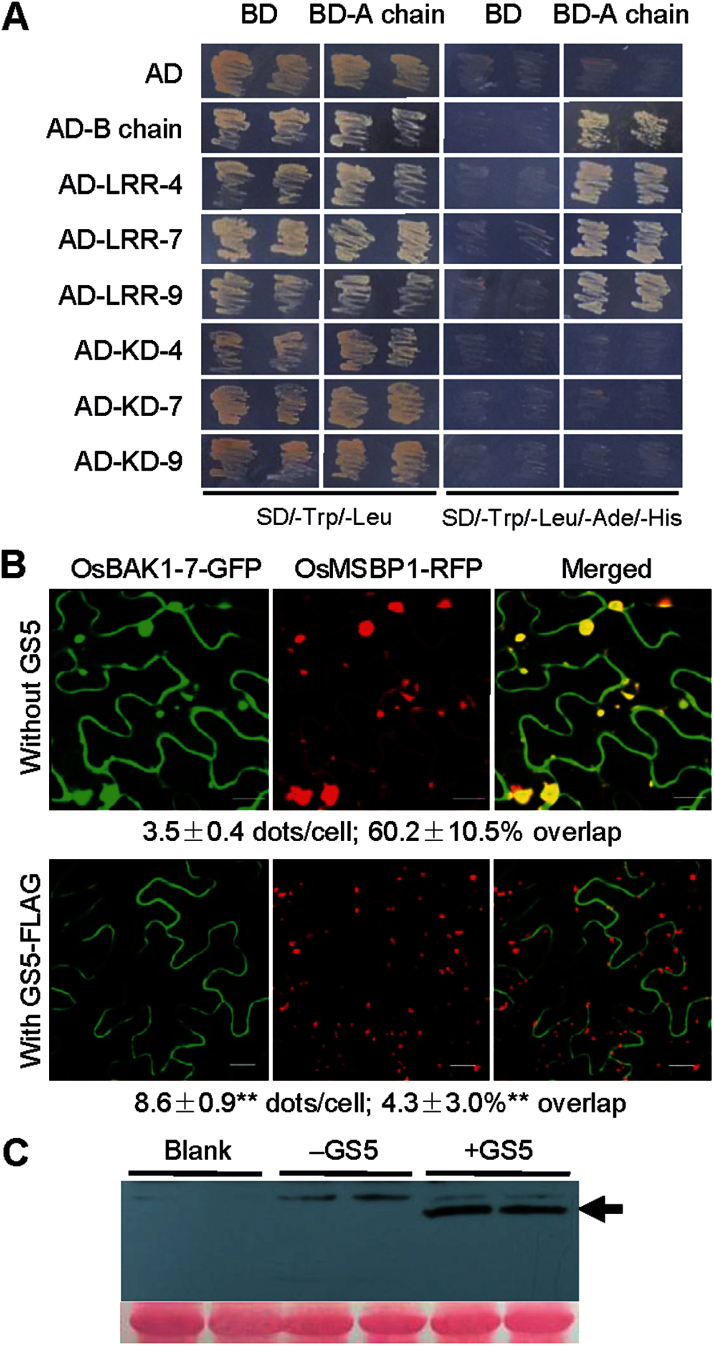
Protein–protein interaction between GS5 and OsBAK1. (A) Yeast two-hybrid assay showing interactions of the A-chain of GS5 (BD-A chain) with the B-chain (AD-B chain) and the extracellular LRR domains of three BAK1 homologues, OsBAK1-4, OsBAK1-7, and OsBAK1-9 (AD-LRR-4, AD-LRR-7, and AD-LRR-9), but not with the GAL4 activation domain (AD) or the kinase domains of OsBAK1 (AD-KD-4, AD-KD-7, and AD-KD-9). BD, GAL4 DNA-binding domain; AD, GAL4 activation domain. (B) Co-expression of OsBAK1-7–GFP and OsMSBP1–RFP in tobacco leaf epidermal cells showing co-localization of the two proteins in the vesicle-like compartments inside the cell (top panel). Co-expression of OsBAK1-7–GFP and OsMSBP1–RFP with GS5-FLAG in tobacco leaf epidermal cells showing the distinct localization of OsBAK1-7–GFP and OsMSBP1–RFP (bottom panel). The experiments were repeated three times, yielding similar results, and representative images are shown. Scales bars=20 μm. The data for the statistical analysis of the fluorescent signals in the cytoplast beneath the images are presented as the mean ±SE (*n* ≥10). **P<0.01, *t*-test. (C) Western blot assay of GS5-FLAG expression in the experiment described in (B) using anti-FLAG antibody. Top panel, western blot assay; bottom panel, Ponceau-stained protein sample; black arrow, full-length GS5-FLAG protein.

To identify interacting proteins, the A-chain of GS5, which included a putative substrate-binding region (Feng and Xue, 2006) (Supplementary Fig. S5 at *JXB* online), was used to screen a yeast two-hybrid library generated using the total RNA from young panicles of 2cm in length from Zhenshan 97. A B-chain of a SCPL protein was identified, indicating that the A-chain folded correctly in yeast and could be used as a bait (Fig. 6A). A homologue of the membrane protein BAK1 was detected. Interactions were also detected between the A-chain of GS5 and the extracellular LRR domains of three BAK1 homologues (Supplementary Fig. S6), but not their intracellular kinase domains or GAL4 activation domain (Fig. 6A).

In *Arabidopsis thaliana*, membrane steroid-binding protein 1 (MSBP1) specifically interacts with the extracellular domain of BAK1 *in vivo* which accelerates the endocytosis of BAK1, resulting in suppressed BR signalling (Song *et al.*, 2009). It was thus suspected that enhanced GS5 expression would competitively inhibit the interaction between OsBAK1 and OsMSBP1 by occupying the extracellular LRR domain of OsBAK1, thus preventing endocytosis of OsBAK1.

To test this hypothesis, the MSBP1 homologue in rice (LOC_Os10g35870) was first obtained by BLAST, and then *P*_*35S*_ *::OsMSBP1-RFP* was constructed and co-expressed with *P*_*35S*_ *::OsBAK1-7-GFP* in tobacco leaf epidermal cells. OsBAK1-7–GFP was localized at the plasma membrane when expressed alone (Fig. 5C). However, when it was co-expressed with OsMSBP1–RFP, a large part of the OsBAK1-7–GFP signal appeared in the vesicle-like compartments, co-localized with OsMSBP1–RFP just as in *A. thaliana* (Fig. 6B, top panel). Next *P*_*35S*_ *::OsBAK1-7-GFP*, *P*_*35S*_ *::OsMSBP1-RFP*, and *P*_*Ubi*_ *::GS5-FLAG* were co-expressed in tobacco leaf epidermal cells, and the expression of GS5-FLAG was detected by western blot using anti-FLAG antibody (Fig. 6C). The results showed that when GS5-FLAG was present, there was no overlap between the subcellular localizations of OsBAK1-7–GFP and OsMSBP1–RFP. The signal of OsBAK1-7–GFP appeared only at the plasma membrane, while OsMSBP1–RFP was found in numerous vesicle–like small compartments (Fig. 6B, bottom panel), which were obviously different from those when co-expressed with OsBAK1-7–GFP only (Fig. 6B, top panel). This suggested that the secreted GS5 protein could competitively interact with the extracellular LRR domains of OsBAK1 on the cell surface, which could explain the correlation between grain size and the expression level of *GS5*.

## Discussion

### The expression of *GS5* is differentially regulated in different tissues

Most of the differences in phenotypes result from variations in protein function or gene expression. In the case of *GS5*, phenotypic variation is due to the expression difference caused by the polymorphisms in the promoter, while both the wide-grain allele *GS5-1* and the narrow-grain allele *GS5-2* encode a functional SCPL protein and higher expression of both proteins produces wider grains. This is similar to the situation of another positive grain-width regulator *GW8/OsSPL16*, whose expression is reduced because of a 10bp deletion in the promoter (Wang *et al.*, 2012). However, *GS5* showed a more complex spatial and temporal expression pattern in the life cycle of rice; it is regulated by multiple elements in the promoter whose polymorphisms caused differential expression of the gene in different tissues. In addition to the two key nucleotides in fractions B (SNP_–1109 and SNP_–1032) of the *GS5* promoter that alter the response of *GS5* to ABA, a number of light-responsive elements are also involved, which regulate activation of transcription and light-induced expression of *GS5* in leaves. Together these variations lead to the result that the *GS5-2* transcript is more abundant in green tissues, while *GS5-1* has a higher expression level in developing panicles.

As a positive regulator of mitosis, differential expression of *GS5* during young panicle development regulates grain size variation. However, the *GS5* transcript in leaves does not affect leaf size, suggesting that *GS5* may have a different cellular function in the leaf. *GS5* also regulates grain filling, which involves the accumulation, distribution, and transportation of a number of substances (Wang *et al.*, 2008; Wu *et al.*, 2008; Ishimaru *et al.*, 2013).

### A proposed model for GS5 regulation of grain size

In this study, it was found that the GS5 protein on the cell surface and OsMSBP1 competitively interact with the extracellular LRR domain of OsBAK1-7, and they have opposite effects on the localization of OsBAK1-7; OsMSBP1 accelerated the endocytosis of OsBAK1-7, whereas GS5 kept OsBAK1-7 on the cell surface. In *A. thaliana*, enhanced expression of MSBP1 suppressed BR signalling by shifting the equilibrium of BAK1 toward endosomes and reducing the BRI1–BAK1 association at the plasma membrane, which can be recovered by overexpressing BAK1 (Song *et al.*, 2009). Furthermore, BR-related genes are shown to have effects on grain weight. Loss-of-function mutants of the genes for BR biosynthesis and the signalling pathway usually have shorter grains (Yamamuro *et al.*, 2000; Hong *et al.*, 2003, 2005; Tanabe *et al.*, 2005; Morinaka *et al.*, 2006; Nakamura *et al.*, 2006). Modification of the endogenous expression level of *OsBAK1*, a member of the same protein family as *OsBAK1-7*, can also alter grain size (Li *et al.*, 2009). BR also regulates grain filling by stimulating the flow of assimilates from the source to the sink (Wu *et al.*, 2008). Transgenic plants expressing a sterol C-22 hydroxylase, which controls BR levels using a promoter active only in the stems, leaves, and roots, produce heavier grains, but the enzyme has no apparent effect on grain weight when expressed in the embryos or endosperms (Wu *et al.*, 2008).

**Fig. 7.**
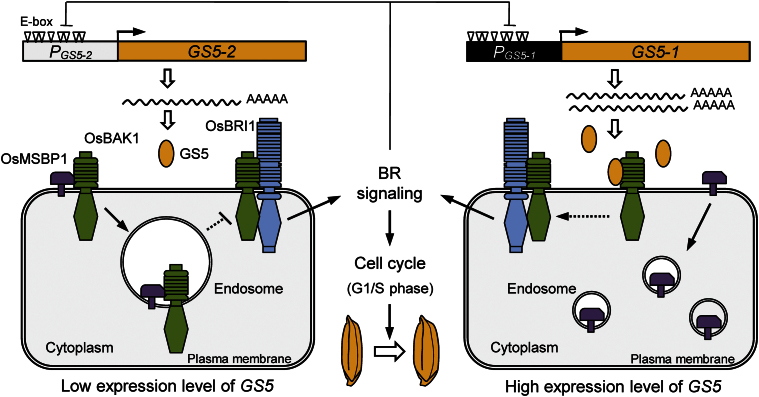
The hypothetical molecular mechanism of GS5 regulation of grain size. The two key SNPs in promoter fraction B result in different expression levels of the *GS5* alleles (*GS5-1* from Zhenshan 97 and *GS5-2* from H94) in developing young panicles. The secreted GS5 protein and the membrane steroid-binding protein 1 homologue (OsMSBP1) can competitively interact with the extracellular leucine-rich repeat (LRR) domain of OsBAK1. When the *GS5* expression level is low, the membrane protein OsBAK1 interacts with OsMSBP1 and the interaction accelerates the endocytosis of OsBAK1, reducing the OsBRI1–OsBAK1 complex at the plasma membrane. With increased expression of *GS5*, the large amount of GS5 protein on the cell surface occupies the extracellular domain of OsBAK1, preventing it from interacting with OsMSBP1 and keeping it at the plasma membrane, thus facilitating the OsBRI1–OsBAK1 interaction. The OsBRI1–OsBAK1 interaction enhances BR signalling, which promotes mitotic division in the lemma/palea, resulting in wider grains, and also feedback suppression of the *GS5* transcription via the E-boxes (indicated by the triangle) in the promoter of *GS5*. (This figure is available in colour at *JXB* online.)

Based on the above results, it was reasonable to assume that the increased expression of the secreted GS5 protein on the cell surface kept OsBAK1 at the plasma membrane, where it could interact with OsBRI1 and enhance BR signalling. Then the enhanced BR signalling would have a dual effect on the GS5-regulated grain size: (i) it would promote the cell cycle thus increasing grain size; but at the same time (ii) it would suppress *GS5* transcription via the E-boxes in the promoter by feedback regulation. The final grain size is thus the outcome of a balance between them (Fig. 7).

Interestingly, there is a secreted SCPL protein, BRS1, which shared high sequence similarity with GS5, involved in BR signalling in *A. thaliana*. The effect of BRS1 is selective: enhanced expression of *BRS1* can suppress the *bri1* extracellular domain mutants, but overexpression in either the wild type or the kinase-dead *bri1* mutant results in no phenotypic alterations (Li *et al.*, 2001), and the degree of suppression of the *bri1* mutant is also positively correlated with the *BRS1* expression level (Zhou and Li, 2005). These studies suggest that BRS1 probably acts at an early step in BR signalling by processing some rate-limiting protein(s), but the actual molecular mechanism is still unclear (Li *et al.*, 2001; Zhou and Li, 2005). In addition to the similar characteristics between GS5 and BRS1, it was also found that overexpression of BRS1 in rice can increase grain width, and the grain width is positively correlated with the BRS1 expression level just as in the case of GS5 (Supplementary Fig. S7 at *JXB* online), strongly suggesting that BRS1 may function similarly to GS5. These results strongly suggest a potential link among GS5, the BR-related pathway, and grain size regulation, although more research is still needed to pinpoint the exact underlying mechanism.

### What makes *GS5* a minor gene for grain size?

*GS5* is a minor QTL controlling grain size; grains of NIL(ZS97) are only 8.7% wider than those of NIL(H94) (Li *et al.*, 2011). The slight variation of grain size stemmed from the limited expression difference caused by the polymorphisms in the promoter region. Since an elevated expression level of *GS5* increases grain size, the question then arises of how much of a grain size increase can be obtained by manipulating *GS5*. Can overexpressing the gene with a stronger promoter further increase the grain size? It is clear from the present results that the answer to this question is no. Overexpression of *GS5* increases the grain width by only ~6% (Table 1), clearly indicating that the GS5 protein has only a limited effect on grain size determination. This is probably because the effect of competitive interaction of the proteins is dependent on the quantity of the other two proteins, and is also subject to feedback regulation of BR signalling. Also, excess GS5 proteins may get stuck in the ER. Therefore, it is not possible to increase grain size beyond a certain range by excess expression of *GS5*.

## Supplementary data

Supplementary data are available at *JXB* online.

Figure S1. Grain size and chalkiness of the *phyB* mutant.

Figure S2. Phylogenetic tree of members of the serine carboxypeptidase-like protein family.

Figure S3. Assay of GS5-FLAG protein in *P*_*Ubi*_ *::GS5-FLAG* transgenic positive plants.

Figure S4. Co-localization assay of GS5 with the endoplasmic reticulum marker inside the cell.

Figure S5. Amino acid alignment of group II serine carboxypeptidase-like proteins.

Figure S6. Phylogenetic tree of the rice BAK1 homologues.

Figure S7. Grain size and leaf size of *P*_*Ubi*_ *:: AtBRS1-FLAG* transgenic plants.

Table S1. Polymorphisms and *cis*-acting elements in the 2kb promoter of *GS5*.

Table S2. Primers used in this work.

Supplementary Data

## References

[CIT0001] Fan C Xing Y Mao H Lu T Han B Xu C Li X Zhang Q 2006 *GS3*, a major QTL for grain length and weight and minor QTL for grain width and thickness in rice, encodes a putative transmembrane protein. Theoretical and Applied Genetics 112, 1164–1171. 16453132 10.1007/s00122-006-0218-1

[CIT0002] Feng Y Xue Q 2006 The serine carboxypeptidase like gene family of rice (*Oryza sativa* L. ssp. *japonica*). Functional and Integrative Genomics 6, 14–24. 15809843 10.1007/s10142-005-0131-8

[CIT0003] Fraser CM Rider LW Chapple C 2005 An expression and bioinformatics analysis of the Arabidopsis serine carboxypeptidase-like gene family. Plant Physiology 138, 1136–1148. 15908604 10.1104/pp.104.057950 PMC1150427

[CIT0004] Gubler F Jacobsen JV 1992 Gibberellin-responsive elements in the promoter of a barley high-pI alpha-amylase gene. The Plant Cell 4, 1435–1441. 1477556 10.1105/tpc.4.11.1435 PMC160230

[CIT0005] He J-X Gendron JM Sun Y Gampala SSL Gendron N Sun CQ Wang Z-Y 2005 BZR1 is a transcriptional repressor with dual roles in brassinosteroid homeostasis and growth responses. Science 307, 1634–1638. 15681342 10.1126/science.1107580 PMC2925132

[CIT0006] Hong Z Ueguchi-Tanaka M Fujioka S Takatsuto S Yoshida S Hasegawa Y Ashikari M Kitano H Matsuoka M 2005 The rice *brassinosteroid-deficient dwarf2* mutant, defective in the rice homolog of Arabidopsis DIMINUTO/DWARF1, is rescued by the endogenously accumulated alternative bioactive brassinosteroid, dolichosterone. The Plant Cell 17, 2243–2254. 15994910 10.1105/tpc.105.030973 PMC1182486

[CIT0007] Hong Z Ueguchi-Tanaka M Umemura K Uozu S Fujioka S Takatsuto S Yoshida S Ashikari M Kitano H Matsuoka M 2003 A rice brassinosteroid-deficient mutant, *ebisu dwarf (d2)*, is caused by a loss of function of a new member of cytochrome P450. The Plant Cell 15, 2900–2910. 14615594 10.1105/tpc.014712 PMC282825

[CIT0008] Huang L Sun Q Qin F Li C Zhao Y Zhou D-X 2007 Down-regulation of a *SILENT INFORMATION REGULATOR2*-related histone deacetylase gene, *OsSRT1*, induces DNA fragmentation and cell death in rice. Plant Physiology 144, 1508–1519. 17468215 10.1104/pp.107.099473 PMC1914135

[CIT0009] Ishimaru K Hirotsu N Madoka Y 2013 Loss of function of the IAA-glucose hydrolase gene TGW6 enhances rice grain weight and increases yield. Nature Genetics 45, 707–711. 23583977 10.1038/ng.2612

[CIT0010] Jacobsen JV Beach LR 1985 Control of transcription of α-amylase and rRNA genes in barley aleurone protoplasts by gibberellin and abscisic acid. Nature 316, 275–277.

[CIT0011] Jefferson RA Kavanagh TA Bevan MW 1987 GUS fusions: beta-glucuronidase as a sensitive and versatile gene fusion marker in higher plants. EMBO Journal 6, 3901–3907. 3327686 10.1002/j.1460-2075.1987.tb02730.x PMC553867

[CIT0012] Lescot M Déhais P Thijs G Marchal K Moreau Y Van de Peer Y Rouzé P Rombauts S 2002 PlantCARE, a database of plant *cis*-acting regulatory elements and a portal to tools for *in silico* analysis of promoter sequences. Nucleic Acids Research 30, 325–327. 11752327 10.1093/nar/30.1.325 PMC99092

[CIT0013] Li D Wang L Wang M Xu Y-Y Luo W Liu Y-J Xu Z-H Li J Chong K 2009 Engineering *OsBAK1* gene as a molecular tool to improve rice architecture for high yield. Plant Biotechnology Journal 7, 791–806. 19754838 10.1111/j.1467-7652.2009.00444.x

[CIT0014] Li J Lease KA Tax FE Walker JC 2001 BRS1, a serine carboxypeptidase, regulates BRI1 signaling in *Arabidopsis thaliana* . Proceedings of the National Academy of Sciences, USA 98, 5916–5921. 10.1073/pnas.091065998 PMC33313 11320207

[CIT0015] Li J Wen J Lease KA Doke JT Tax FE Walker JC 2002 BAK1, an *Arabidopsis* LRR receptor-like protein kinase, interacts with BRI1 and modulates brassinosteroid signaling. Cell 110, 213–222. 12150929 10.1016/s0092-8674(02)00812-7

[CIT0016] Li Y Fan C Xing Y 2011 Natural variation in *GS5* plays an important role in regulating grain size and yield in rice. Nature Genetics 43, 1266–1269. 22019783 10.1038/ng.977

[CIT0017] Lin YJ Zhang Q 2005 Optimising the tissue culture conditions for high efficiency transformation of indica rice. Plant Cell Reports 23, 540–547. 15309499 10.1007/s00299-004-0843-6

[CIT0018] Mao H Sun S Yao J Wang C Yu S Xu C Li X Zhang Q 2010 Linking differential domain functions of the GS3 protein to natural variation of grain size in rice. Proceedings of the National Academy of Sciences, USA 107, 19579–19584. 10.1073/pnas.1014419107 PMC2984220 20974950

[CIT0019] Miao Y Jiang L 2007 Transient expression of fluorescent fusion proteins in protoplasts of suspension cultured cells. Nature Protocols 2, 2348–2353. 17947977 10.1038/nprot.2007.360

[CIT0020] Morinaka Y Sakamoto T Inukai Y Agetsuma M Kitano H Ashikari M Matsuoka M 2006 Morphological alteration caused by brassinosteroid insensitivity increases the biomass and grain production of rice. Plant Physiology 141, 924–931. 16714407 10.1104/pp.106.077081 PMC1489896

[CIT0021] Nakamura A Fujioka S Sunohara H 2006 The role of *OsBRI1* and its homologous genes, *OsBRL1* and *OsBRL3*, in rice. Plant Physiology 140, 580–590. 16407447 10.1104/pp.105.072330 PMC1361325

[CIT0022] Nam KH Li J 2002 BRI1/BAK1, a receptor kinase pair mediating brassinosteroid signaling. Cell 110, 203–212. 12150928 10.1016/s0092-8674(02)00814-0

[CIT0023] Sambrook J Fritsch EF Maniatis T 1989 Molecular cloning: a laboratory manual , 2nd edn. Cold Spring Harbor, NY: Cold Spring Harbor Laboratory Press.

[CIT0024] Shomura A Izawa T Ebana K Ebitani T Kanegae H Konishi S Yano M 2008 Deletion in a gene associated with grain size increased yields during rice domestication. Nature Genetics 40, 1023–1028. 18604208 10.1038/ng.169

[CIT0025] Skriver K Olsen FL Rogers JC Mundy J 1991 *cis*-acting DNA elements responsive to gibberellin and its antagonist abscisic acid. Proceedings of the National Academy of Sciences, USA 88, 7266–7270. 10.1073/pnas.88.16.7266 PMC52275 1831269

[CIT0026] Song L Shi Q-M Yang X-H Xu Z-H Xue H-W 2009 Membrane steroid-binding protein 1 (MSBP1) negatively regulates brassinosteroid signaling by enhancing the endocytosis of BAK1. Cell Research 19, 864–876. 19532123 10.1038/cr.2009.66

[CIT0027] Song X-J Huang W Shi M Zhu M-Z Lin H-X 2007 A QTL for rice grain width and weight encodes a previously unknown RING-type E3 ubiquitin ligase. Nature Genetics 39, 623–630. 17417637 10.1038/ng2014

[CIT0028] Sparkes IA Runions J Kearns A Hawes C 2006 Rapid, transient expression of fluorescent fusion proteins in tobacco plants and generation of stably transformed plants. Nature Protocols 1, 2019–2025. 17487191 10.1038/nprot.2006.286

[CIT0029] Sun Q Zhou D-X 2008 Rice jmjC domain-containing gene *JMJ706* encodes H3K9 demethylase required for floral organ development. Proceedings of the National Academy of Sciences, USA 105, 13679–13684. 10.1073/pnas.0805901105 PMC2533249 18765808

[CIT0030] Tanabe S Ashikari M Fujioka S 2005 A novel cytochrome P450 is implicated in brassinosteroid biosynthesis via the characterization of a rice dwarf mutant, *dwarf11*, with reduced seed length. The Plant Cell 17, 776–790. 15705958 10.1105/tpc.104.024950 PMC1069698

[CIT0031] Tripathi L Sowdhamini R 2006 Cross genome comparisons of serine proteases in Arabidopsis and rice. BMC Genomics 7, 200. 16895613 10.1186/1471-2164-7-200 PMC1560137

[CIT0032] Wang E Wang J Zhu X 2008 Control of rice grain-filling and yield by a gene with a potential signature of domestication. Nature Genetics 40, 1370–1374. 18820698 10.1038/ng.220

[CIT0033] Wang S Wu K Yuan Q 2012 Control of grain size, shape and quality by *OsSPL16* in rice. Nature Genetics 44, 950–954. 22729225 10.1038/ng.2327

[CIT0034] Washio K Ishikawa K 1994 Organ-specific and hormone-dependent expression of genes for serine carboxypeptidases during development and following germination of rice grains. Plant Physiology 105, 1275–1280. 7972496 10.1104/pp.105.4.1275 PMC159459

[CIT0035] Weng J Gu S Wan X 2008 Isolation and initial characterization of *GW5*, a major QTL associated with rice grain width and weight. Cell Research 18, 1199–1209. 19015668 10.1038/cr.2008.307

[CIT0036] Weng X Wang L Wang J Hu Y Du H Xu C Xing Y Li X Xiao J Zhang Q 2014 *Grain number, plant height, and heading date 7* is a central regulator of growth, development, and stress response. Plant Physiology 164, 735–747. 24390391 10.1104/pp.113.231308 PMC3912102

[CIT0037] Wu C-y Trieu A Radhakrishnan P 2008 Brassinosteroids regulate grain filling in rice. The Plant Cell 20, 2130–2145. 18708477 10.1105/tpc.107.055087 PMC2553602

[CIT0038] Yamamuro C Ihara Y Wu X Noguchi T Fujioka S Takatsuto S Ashikari M Kitano H Matsuoka M 2000 Loss of function of a rice *brassinosteroid insensitive1* homolog prevents internode elongation and bending of the lamina joint. The Plant Cell 12, 1591–1606. 11006334 10.1105/tpc.12.9.1591 PMC149072

[CIT0039] Ye R Zhou F Lin Y 2012 Two novel positive cis-regulatory elements involved in green tissue-specific promoter activity in rice (*Oryza sativa* L ssp.). Plant Cell Reports 31, 1159–1172. 22388917 10.1007/s00299-012-1238-8

[CIT0040] Yin Y Vafeados D Tao Y Yoshida S Asami T Chory J 2005 A new class of transcription factors mediates brassinosteroid-regulated gene expression in *Arabidopsis* . Cell 120, 249–259. 15680330 10.1016/j.cell.2004.11.044

[CIT0041] Yu X Li L Zola J 2011 A brassinosteroid transcriptional network revealed by genome-wide identification of BESI target genes in *Arabidopsis thaliana* . The Plant Journal 65, 634–646. 21214652 10.1111/j.1365-313X.2010.04449.x

[CIT0042] Zhou A Li J 2005 Arabidopsis BRS1 is a secreted and active serine carboxypeptidase. Journal of Biological Chemistry 280, 35554–35561. 16123046 10.1074/jbc.M503299200

