# Supplementary material for: Differential expression of GS5 regulates grain size in rice
Source: J Exp Bot. 2015 Feb 24;66(9):2611–23. doi: 10.1093/jxb/erv058 (PMC4986870; doi:10.1093/jxb/erv058)
Supplement: Supplementary Data [file supp_66_9_2611__index.html]

Differential expression of GS5 regulates grain size in rice — Differential expression of GS5 regulates grain size in rice — Supplementary Data 

# Differential expression of *GS5* regulates grain size in rice

## Supplementary Data

Data files

**Files in this Data Supplement:**

- Supplementary Data - Supplementary Data
