# Supplementary material for: Differential expression of GS5 regulates grain size in rice
Source: J Exp Bot. 2015 Feb 24;66(9):2611–23. doi: 10.1093/jxb/erv058 (PMC4986870; doi:10.1093/jxb/erv058)
Supplement: Supplementary Data [file supp_erv058_jexbot137109_file001.pdf]

Journal of Experimental Botany

Differential expression of *GS5* regulates grain size in rice

Chunjue Xu, Yu Liu, Yibo Li, Xiaodong Xu, Caiguo Xu, Xianghua Li, Jinghua Xiao and Qifa Zhang[1]

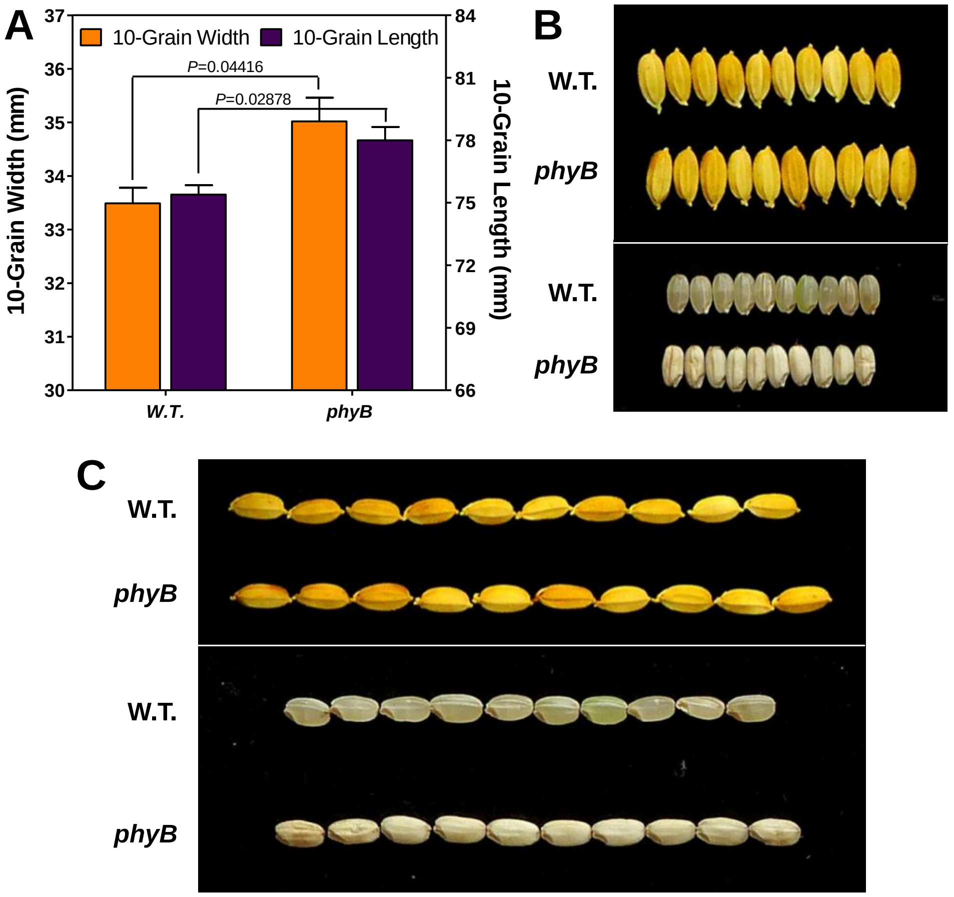

Figure S1. Grain size and chalkiness of *phyB* mutant. (A) Significant difference in grain size between *phyB* mutant and wild type (W.T.). All data are presented as mean ± SE (n≥3). *P* values were calculated using Student's *t*-test. (B) Grain width and chalkiness of *phyB* mutant and wild type. (C) Grain length and chalkiness of *phyB* mutant and wild type.

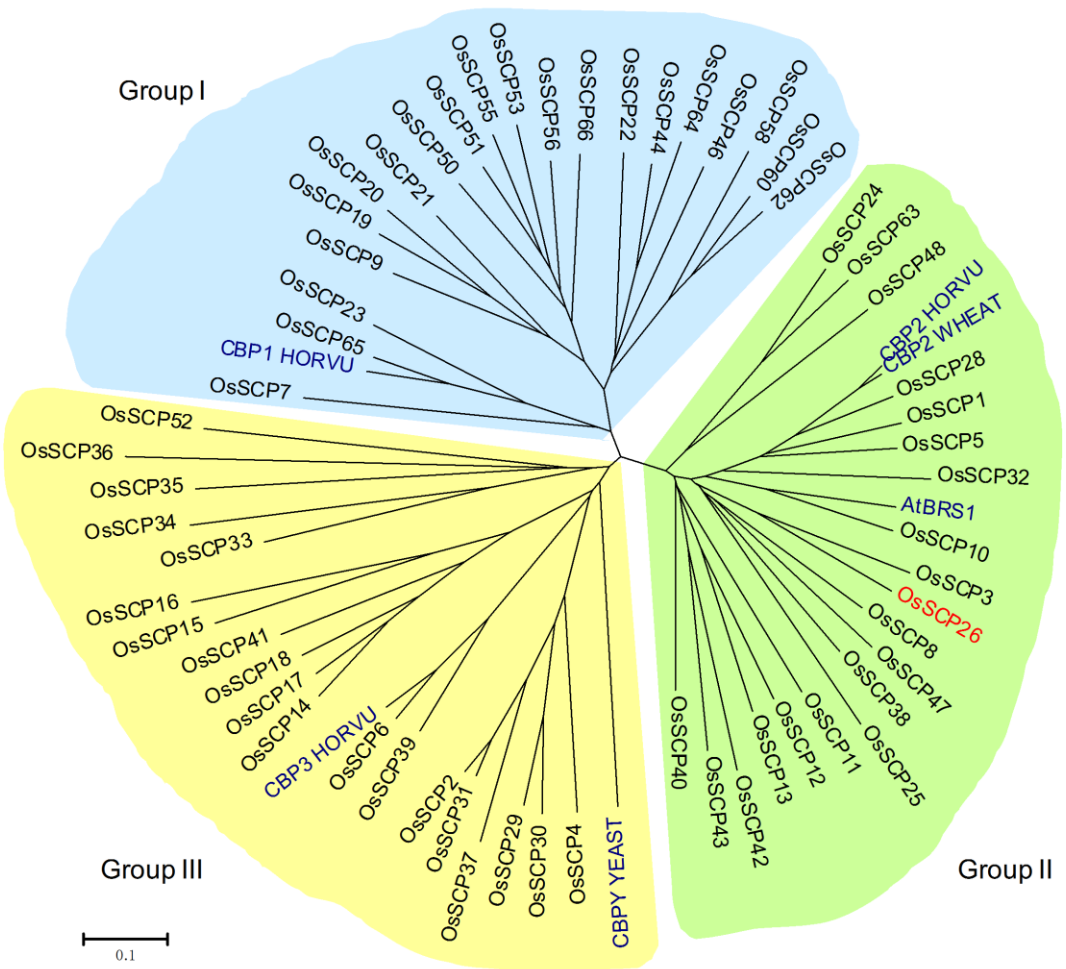

Figure S2. Phylogenetic tree of members of the serine carboxypeptidase-like protein family. Full-length protein sequences of OsSCPLs were downloaded from http://rice.plantbiology.msu.edu/ca/gene_fams/27_76.shtml and aligned with ClustalX and calculated with MEGA 3.1. *CBP1 HORVU* (NCBI accession NO. P07519), *CBP2 HORVU* (NCBI accession NO. P08818), *CBP3 HORVU* (NCBI accession NO. P21529) and *CBP2 WHEAT* (NCBI accession NO. P08819) were used to indicate the three groups of SCPL. *GS5* (*OsSCP26*) and *AtBRS1* (NCBI accession NO. NP_194790) belonging to SCPL group II; *CBPY YEAST* (NCBI accession NO. P00729) belongs to SCPL group III.

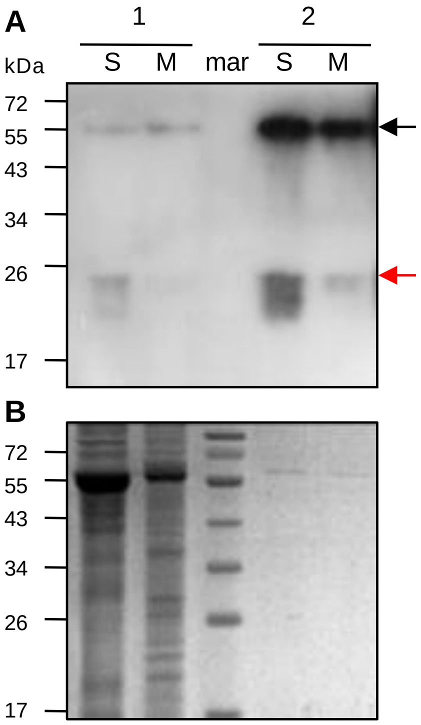

Figure S3. Assay of GS5-FLAG protein in *P_{Ubi}::GS5-FLAG* transgenic positive plants. (A) Western blot assay of GS5-FLAG transgenic plants using anti-FLAG antibody showing the cleavage of GS5-FLAG in *P_{Ubi}::GS5-FLAG* transgenic positive plants into two chains (A and B). 1, Total protein extracted from transgenic positive plants; 2, Eluted proteins after anti-FLAG M2 agarose affinity purification; mar, Marker; S, Soluble protein sample; M, solubilized membrane protein sample; black arrow, full-length GS5-FLAG protein; red arrow, the C-terminal of GS5, B chain-FLAG peptide. (B) Coomassie Blue-stained protein samples of (A).

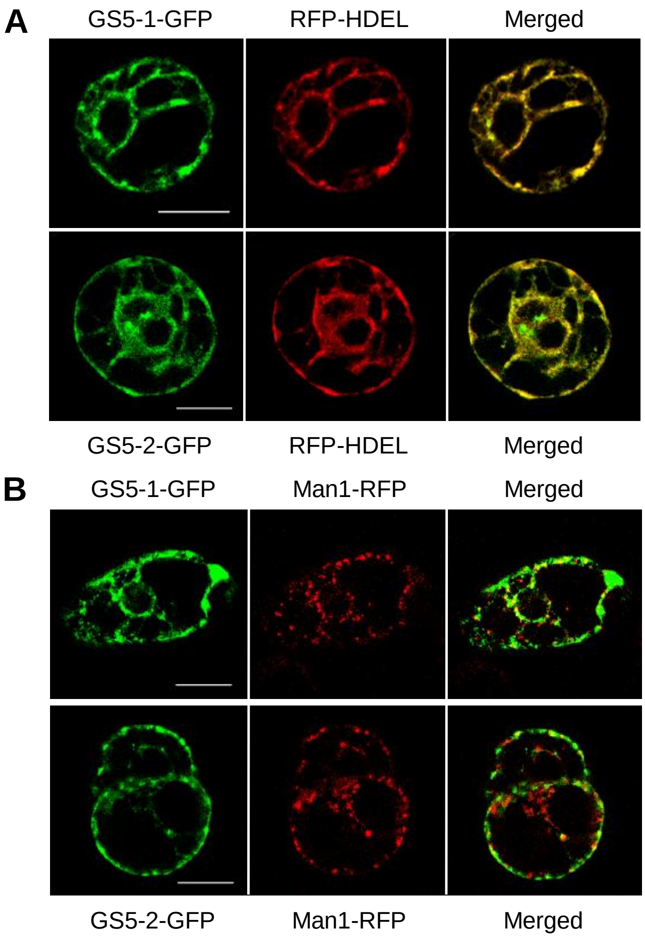

Figure S4. Co-localization assay of GS5 with endoplasmic reticulum marker inside the cell. (A) Co-expression of GS5-GFP and endoplasmic reticulum marker RFP-HDEL in tobacco BY-2 protoplasts showing co-localization of the two proteins. (B) Co-expression of GS5-GFP and *cis*-Golgi marker Man1-RFP in tobacco BY-2 protoplasts showing distinct localization of the two proteins. The experiments were repeated three times, yielding similar results, and representative images are shown. Scales bars = 20 μm.

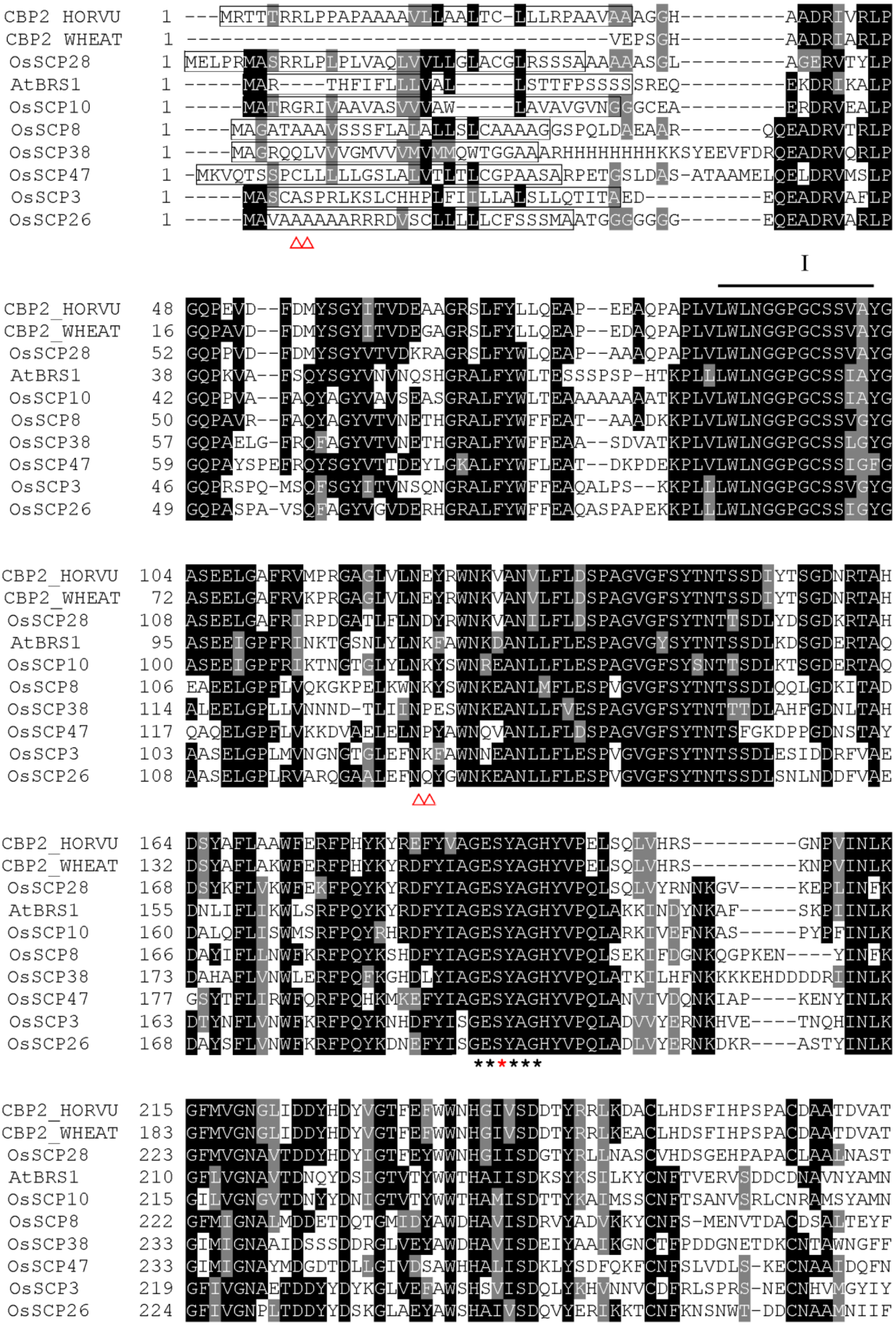

CBP2_HORVU 1 ---MRTTTRRLPPAPAAAAVLLAALTC-LLLRPAAVAAAGGH---------AADRIVRLP
CBP2_WHEAT 1 ------------------------------------VEPSGH---------AADRIARLP
OsSCP28 1 MELPRMASRRLPLPLVAQLVVLLGLACGLRSSSAAAAAASGL---------AGERVTYLP
AtBRS1 1 -----MAR----THFIFLLVAL-----LSTTFPSSSSSREQ---------EKDRIKALP
OsSCP10 1 -----MATRGRIVAAVASVVVAW-----LAVAVGVNGGGCEA---------ERDRVEALP
OsSCP8 1 ----MAGATAAAVSSSFLALALLSLCAAAAGGSPQLDAEAAR-------QQEADRVTRLP
OsSCP38 1 ----MAGRQQLVVVGMVVVMVMMQWTGGAAARHHHHHHHHKKSYEEVFDRQEADRVQRLP
OsSCP47 1 -MKVQTSSPCLLLLLGSLALVTLTLCGPAASARPETGSLDAS-ATAAMELQELDRVMSLP
OsSCP3 1 -----MASCASPRLKSLCHHPLFILLLALSLLQTITAED----------EQEADRVAFLP
OsSCP26 1 -----MAVAAAAAARRRDVSCLLLLLCFSSSMAATGGGGGGG-------EQEADRVARLP
I
CBP2_HORVU 48 GQPEVD--FDMYSGYITVDEAAGRSLFYLLQEAP--EEAQPAPLVLWLNGGPGCSSVAYG
CBP2_WHEAT 16 GQPAVD--FDMYSGYITVDEGAGRSLFYLLQEAP--EDAQPAPLVLWLNGGPGCSSVAYG
OsSCP28 52 GQPPVD--FDMYSGYVTVDKRAGRSLFYWLQEAP--AAAQPAPLVLWLNGGPGCSSVAYG
AtBRS1 38 GQPKVA--FSQYSGYVNVNQSHGRALFYWLTESSSPSP-HTKPLLLWLNGGPGCSSIAYG
OsSCP10 42 GQPPVA--FAQYAGYVAVSEASGRALFYWLTEAAAAAAATKPLVLWLNGGPGCSSIAYG
OsSCP8 50 GQPAVR--FAQYAGYVTVNETHGRALFYWFFEAT--AAADKKPLVLWLNGGPGCSSVGYG
OsSCP38 57 GQPAELG-FRQFAGYVTVNETHGRALFYWFFEAA--SDVATKPLVLWLNGGPGCSSLGYG
OsSCP47 59 GQPAYSPEFRQYSGYVTTDEYLGKALFYWFLEAT--DKPDEKPLVLWLNGGPGCSSIGFG
OsSCP3 46 GQPRSPQ-MSQFSGYITVNSQNGRALFYWFFEAQALPS--KKPLLLWLNGGPGCSSVGYG
OsSCP26 49 GQPASPA-VSQFAGYVGVDERHGRALFYWFFEAQASPAPEKKPLLLWLNGGPGCSSIGYG
CBP2_HORVU 104 ASEELGAFRVMPRGAGLVLNEYRWNKVANVLFLDSPAGVGFSYTNTSSDIYTSGDNRTAH
CBP2_WHEAT 72 ASEELGAFRVKPRGAGLVLNEYRWNKVANVLFLDSPAGVGFSYTNTSSDIYTSGDNRTAH
OsSCP28 108 ASEELGAFRIRPDGATLFLNDYRWNKVANILFLDSPAGVGFSYTNTTSDLYDSGDKRTAH
AtBRS1 95 ASEEIGPFRINKTGSNLYLNKFAWNKDANLLFLESPAGVGYSYTNTSSDLKDSGDERTAQ
OsSCP10 100 ASEEIGPFRIKTNGTGLYLNKYSWNREANLLFLESPAGVGFSYSNTTSDLKTSGDERTAQ
OsSCP8 106 EAEELGPFLVQKGKPELKWNKYSWNKEANLMFLESPVGVGFSYTNTSSDLQQLGDKITAD
OsSCP38 114 ALEELGPLLVNNND-TLIINPESWNKEANLLFVESPAGVGFSYTNTTTDLAHFGDNLTAH
OsSCP47 117 QAQELGPFLVKKDVAELELNPYAWNQVANLLFLDSPAGVGFSYTNTSFGKDPPGDNSTAY
OsSCP3 103 AASELGPLMVNGNGTGLEFNKFAWNNEANLLFLESPVGVGFSYTNTSSDLESIDDRFVAE
OsSCP26 108 AASELGPLRVARQGAALEFNQYGWNKEANLLFLESPVGVGFSYTNTSSDLSNLNDDFVAE
CBP2_HORVU 164 DSYAFLAAWFERFPHYKYREFYVAGESYAGHYVPELSQLVHRS---------GNPVINLK
CBP2_WHEAT 132 DSYAFLAKWFERFPHYKYRDFYIAGESYAGHYVPELSQLVHRS---------KNPVINLK
OsSCP28 168 DSYKFLVKWFEKFPQYKYRDFYIAGESYAGHYVPQLSQLVYRNNKGV-----KEPLINFK
AtBRS1 155 DNLIFLIKWLSRFPQYKYRDFYIAGESYAGHYVPQLAKKINDYNKAF-----SKPIINLK
OsSCP10 160 DALQFLISWMSRFPQYRHRDFYIAGESYAGHYVPQLARKIVEFNKAS-----PYPFINLK
OsSCP8 166 DAYIFLLNWFKRFPQYKSHDFYIAGESYAGHYVPQLSEKIFDGNKQGPKEN----YINFK
OsSCP38 173 DAHAFLVNWLERFPQFKGHDLYIAGESYAGHYVPQLATKILHFNKKKKEHDDDDRIINLK
OsSCP47 177 GSYTFLIRWFQRFPQHKMKEFYIAGESYAGHYVPQLANVIVDQNKIAP----KENYINLK
OsSCP3 163 DTYNFLVNWFKRFPQYKNHDFYISGESYAGHYVPQLADVVYERNKHVE----TNQHINLK
OsSCP26 168 DAYSFLVNWFKRFPQYKDNEFYISGESYAGHYVPQLADLVYERNKDKR----ASTYINLK
******
CBP2_HORVU 215 GFMVGNGLIDDYHDYVGTFEFWWNHGIVSDDTYRRLKDACLHDSFIHPSPACDAATDVAT
CBP2_WHEAT 183 GFMVGNGLIDDYHDYVGTFEFWWNHGIVSDDTYRRLKEACLHDSFIHPSPACDAATDVAT
OsSCP28 223 GFMVGNAVTDDYHDYIGTFEYWWNHGIISDGTYRLLNASCVHDSGEHPAPACLAAINAST
AtBRS1 210 GFLVGNAVTDNQYDSIGTVTYWWTHAIISDKSYKSILKYCNFTVERVSDDCDNAVNYAMN
OsSCP10 215 GILVGNGVTDNYYDNIGTVTYWWTHAMISDTTYKAIMSSCNFTSANVSRLCNRAMSYAMN
OsSCP8 222 GFMIGNALMDDETDQTGMIDYAWDHAVISDRVYADVKKYCNFS-MENVTDACDSALTEYF
OsSCP38 233 GIMIGNAAIDSSSDDRGLVEYAWDHAVISDEIYAAIKGNCTFPDDGNETDKCNTAWNGFF
OsSCP47 233 GIMIGNAYMDGDTDLLGIVDSAWHHALISDKLYSDFQKFCNFSLVDLS-KECNAAIDQFN
OsSCP3 219 GFIVGNAETDDYYDYKGLVEFAWSHSVISDQLYKHVNNVCDFRLSPRS-NECNHVMGYIY
OsSCP26 224 GFIVGNPLTDDYYDSKGLAEYAWSHAIVSDQVYERIKKTCNFKNSNWT-DDCNAAMNIIF

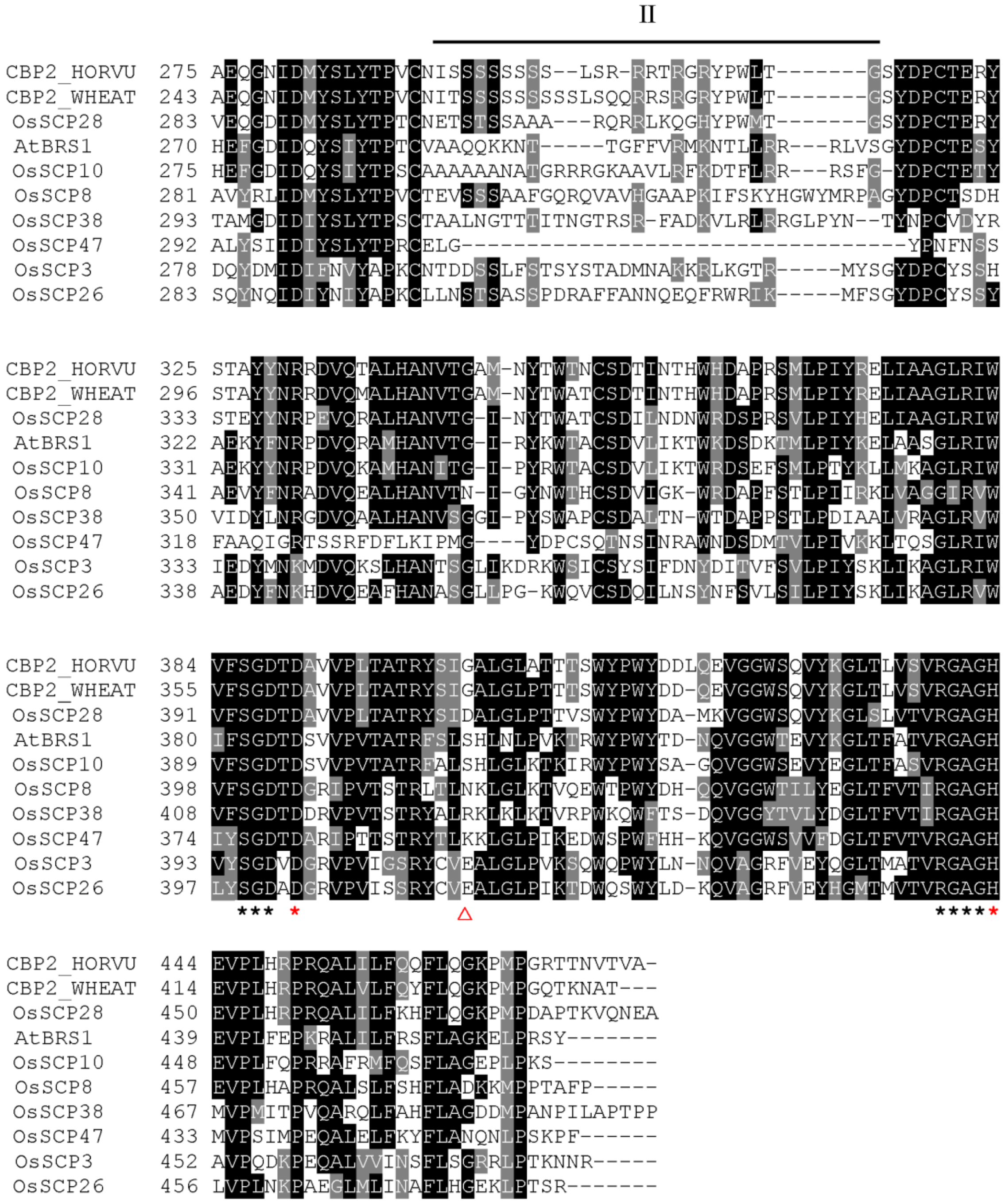

Figure S5. Amino acid alignment of group II serine carboxypeptidase-like proteins. The boxed amino acids at the N terminus represent the putative signal peptide. I and II indicate the putative substrate binding region and linker peptide respectively. The A-chain is between signal peptide and linker peptide, and B-chain is downstream of the linker peptide. The red triangles indicate the different residues between GS5-1 and GS5-2 (OsSCP26). The red asterisks indicate the Ser-Asp-His catalytic triad of SCPL, and the black asterisks mark the residues essential for catalysis.

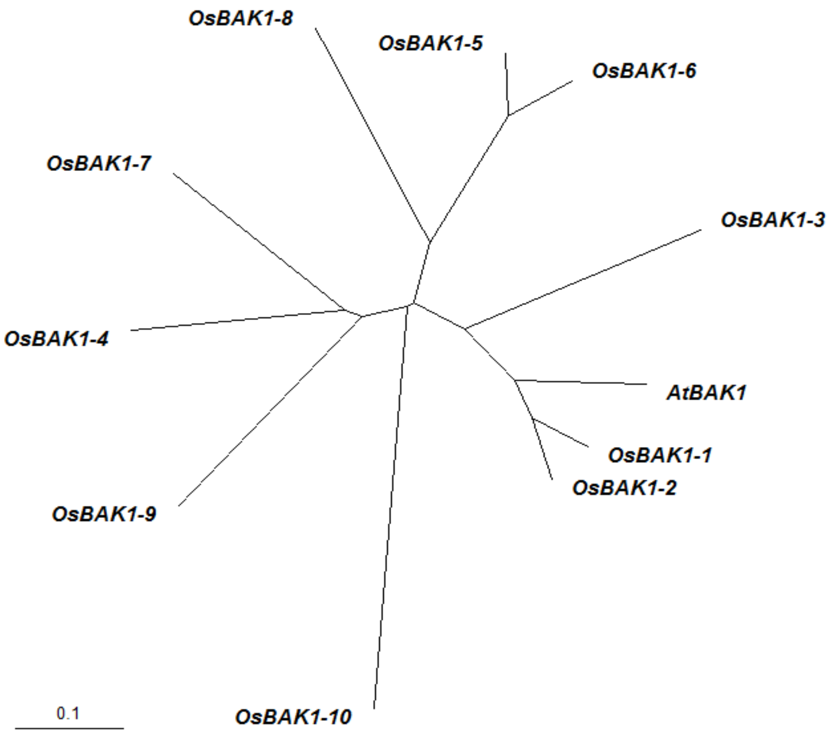

Figure S6. Phylogenetic tree of the rice BAK1 homologues. Full-length protein sequences of the rice BAK1 homologues were obtained by BLAST using the AtBAK1 (NCBI accession NO. NM_119497) sequence from http://rice.plantbiology.msu.edu/index.shtml, aligned with ClustalX and calculated with MEGA 3.1. OsBAK1-1, LOC_Os08g07760; OsBAK1-2, LOC_Os04g38480; OsBAK1-3, LOC_Os06g12120; OsBAK1-4, LOC_Os01g07630; OsBAK1-5, LOC_Os02g18320; OsBAK1-6, LOC_Os03g49620; OsBAK1-7, LOC_Os06g16330; OsBAK1-8, LOC_Os11g39370; OsBAK1-9, LOC_Os02g14120; and OsBAK1-10, LOC_Os03g16010.

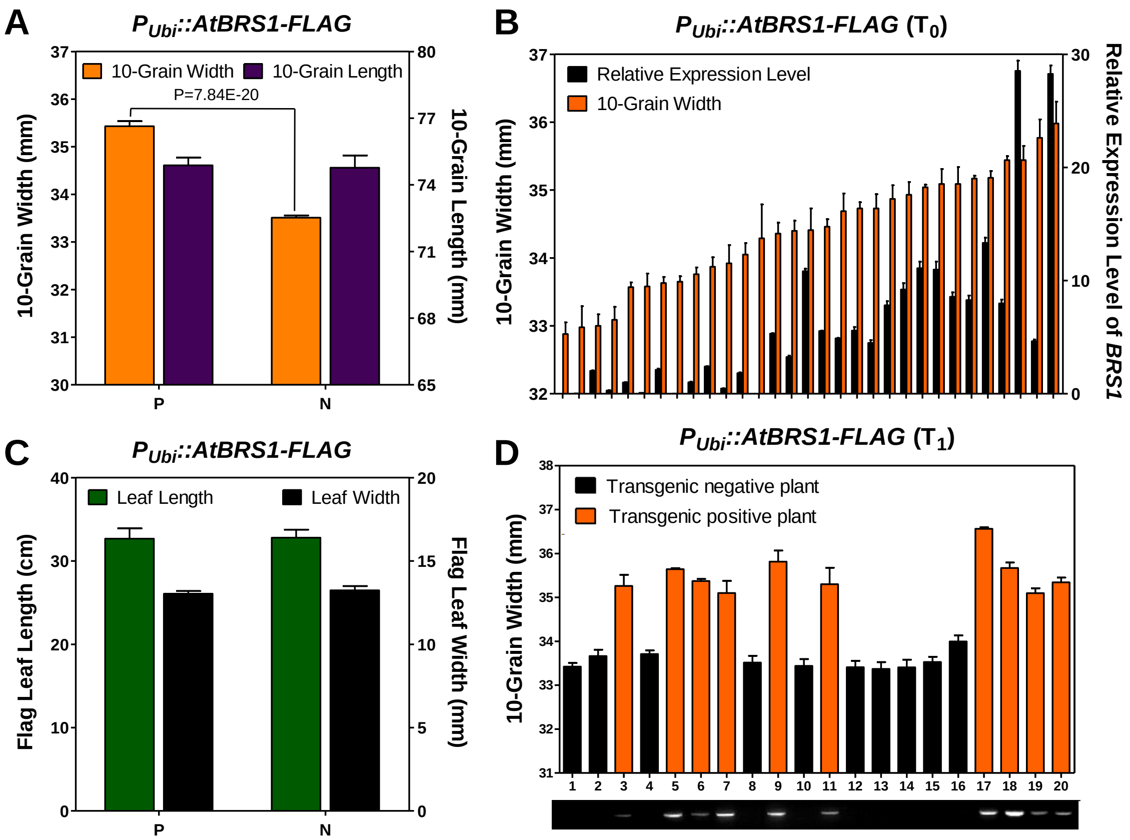

Figure S7. Grain size and leaf size of P_{Ubi}:: *AtBRS1-FLAG* transgenic plants. (A) Grain width and grain length of T1 transgenic rice plants overexpressing *AtBRS1*. P, transgenic positive plants; N, transgenic negative plants. (B) Co-segregation of grain width with expression level of *AtBRS1* in T0 transgenic rice plants. (C) Flag leaf width and length of T1 transgenic rice plants overexpressing *AtBRS1*. P, transgenic positive plants; N, transgenic negative plants. (D) Co-segregation of grain width with *AtBRS1* genotype in T1 transgenic rice plants. All data are presented as mean ± SE (n≥3). *P* values were calculated using Student's *t*-test.

Table S1. Polymorphisms and *cis*-acting elements in the 2-kb promoter of *GS5*.

Position	SNPs/InDels *GS5-1*	SNPs/InDels *GS5-2*	Sequence	*Cis*-acting elements	Organism	Function
–1961	/	/	CAANNNNATC	circadian	*Lycopersicon esculentum*	Circadian control
–1723	/	/	ATTAAT	Box4	*Petroselinum crispum*	Light responsiveness
–1656	/	/	AATCTAATCT	ATCT-motif	*Arabidopsis thaliana*	Light responsiveness
–1630	/	/	CAACTG	MBS	*Arabidopsis thaliana*	MYB binding site, drought-inducibility
				E-box	*Arabidopsis thaliana*	Brassinosteroid responsiveness
–1620	/	/	GGAGATG	GAG-motif	*Hordeum vulgare*	part of a light responsive element
–1595	C	T	/	/	/	/
–1539	/	/	CATATG	E-box	*Arabidopsis thaliana*	Brassinosteroid responsiveness
–1518	/	/	CACGTG	ABRE	*Arabidopsis thaliana*	Abscisic acid responsiveness
				G-box	*Arabidopsis thaliana*	Light responsiveness
				E-box	*Arabidopsis thaliana*	Brassinosteroid responsiveness
–1514	/	/	TGACG	TGACG-motif	*Hordeum vulgare*	MeJA-responsiveness
–1489	/	/	CCGTCC	A-box	*Petroselinum crispum*	*cis*-acting regulatory element
				CCGTCC-box	*Arabidopsis thaliana*	Meristem specific activation
–1476	-	A	/	/	/	/
–1466	T	A	/	/	/	/
–1464	T	G	/	/	/	/
–1463	A	G	/	/	/	/
–1391	G	A	/	/	/	/
–1376	/	/	ATTAAT	Box4	*Petroselinum crispum*	Light responsiveness
–1345	/	/	ATTTTCTTCA	TC-rich repeats	*Nicotiana tabacum*	Defense and stress responsiveness
–1314	/	/	CGGTCA	MBS	*Zea mays*	MYB Binding Site
–1303	A	G	/	/	/	/
–1281	T	-	/	/	/	/
–1242	/	/	TGACG	TGACG-motif	*Hordeum vulgare*	MeJA-responsiveness
–1223	/	/	CACTTG	E-box	*Arabidopsis thaliana*	Brassinosteroid responsiveness
–1218	/	/	GTCAT	Skn-1-motif	*Oryza sativa*	Endosperm expression
–1204	/	/	CCG/ACCC	Spl	*Zea mays*	Light responsive element
–1164	/	/	CACTTG	E-box	*Arabidopsis thaliana*	Brassinosteroid responsiveness
–1159	/	/	GTCAT	Skn-1-motif	*Oryza sativa*	Endosperm expression
–1142	/	/	CATTTG	E-box	*Arabidopsis thaliana*	Brassinosteroid responsiveness
–1109	C	T	/	/	/	/
–1096	/	/	CAATTG	E-box	*Arabidopsis thaliana*	Brassinosteroid responsiveness
–1088	/	/	AAACAGA	GARE-motif	*Brassica oleracea*	Gibberellin-responsive element
–1076	A	G	/	/	/	/
–1032	A	T	/	/	/	/
–1015	G	T	/	/	/	/

Continuing Table S1

–1013	/	/	CAGATG	E-box	*Arabidopsis thaliana*	Brassinosteroid responsiveness
–895		G	GATAGGA	GATA-motif	*Arabidopsis thaliana*	part of a light responsive element
	A		GATAAGATT	I-box	*Arabidopsis thaliana*	part of a light responsive element
–879	/	/	TTTCAAA	BoxI	*Pisum sativum*	Light responsive element
–847	T	-	/	/	/	/
–825		T	TCTTAC	TCT-motif	*Arabidopsis thaliana*	part of a light responsive element
	C		/	/	/	/
–788		C	CCG/ACCC	Spl	*Zea mays*	Light responsive element
	A		/	/	/	/
–756	/	/	ATTAAT	Box4	*Petroselinum crispum*	Light responsiveness
–710	/	/	GTCAT	Skn-1-motif	*Oryza sativa*	Endosperm expression
–671		G	TGACG	TGACG-motif	*Hordeum vulgare*	MeJA-responsiveness
	A		/	/	/	/
–409	/	/	AAACAGA	GARE-motif	*Brassica oleracea*	Gibberellin-responsive element
–375	/	/	TAACTG	MBS	*Arabidopsis thaliana*	MYB binding site, drought-inducibility
–371	/	/	TGAGTCA	GCN4-motif	*Oryza sativa*	Endosperm expression
–368	/	/	GTCAT	Skn-1-motif	*Oryza sativa*	Endosperm expression
–326	-	C	/	/	/	/
–325	-	A	/	/	/	/
–324	-	A	/	/	/	/
–323	-	A	/	/	/	/
–225	/	/	ATTTTCTTCA	TC-rich repeats	*Nicotiana tabacum*	Defense and stress responsiveness
–100	/	/	TTTCTTCTCT	5'UTR Py-rich stretch	*Lycopersicon esculentum*	Conferring high transcription levels

-, single nucleotide deletion; /, not detected.

Table S2. Primers used in this work.

Name	Sequence
**Expression analysis**	
GS5qF	AGTGGACTGCTTCCAGGGAAG
GS5qR	CACGCAGTACCGAGAACTGA
Act1F	TGCTATGTACGTCGCCATCCAG
Act1R	AATGAGTAACCACGCTCCGTCA
**Promoter constructs**	
PAF	AAGCTTTCGTATGGAATTACCTAGCTATGCA
PBF	AAGCTTTTGACGAGAGGCAAGGACCCCA
PCF	AAGCTTGATGGGAGAGATAGATAGACTC
PDF	AAGCTTTGCAACCCGGATCTGGACAGA
PDR	GGATCCTGCTAACCCGTGGTGCCGAGCTCTAGC
–1109mutF	ACACAGGATTTTGACAGGAATAC
–1109mutR	GTATTCCTGTCAAAATCCTGTGT
–1032mutF	TGATTGGACCACTGAAAAAAC
–1032mutR	GTTTTTTCAGTGGTCCAATCA
–825mutF	CTCCAAAATTCTTACATTTTTTCT
–825mutR	AGAAAAAATGTAAGAATTTTGGAG
B_GAmutF	ACAATTGTAACCAAGAGGATTTGTA
B_GAmutR	TACAAATCCTCTTGGTTACAATTGT
C_LIGHTmutF	CTCCAAAATCCTTACATTTTTTC
C_LIGHTmutR	GAAAAAATGTAAGGATTTTGGAG
**cDNA constructs**	
GS5cDNAF	GGTACCTAGCTAGAGCTCGGCACCACGGGTTAGCAA
GS5cDNAR	GGATCCTCTGCTTGTCGGAAGCTTCTCACCATGAAG
OsBAK1-7cDNAF	GGTACCTACCTGAGCTCAGCAATGGA
OsBAK1-7cDNAR	GTTAACTCTGGGGCCGGAGAGCTC
OsMSBP1cDNAF	CTCGAGATGGCGGCGGCGGTGGCGGA
OsMSBP1cDNAR	GGATCCACTTTCTTTGGCGCCTTCATCAGGTGCA
GS5AchainF	GAATTCGGGATCCATATGGAGCAGGAGGCTGACAGGGT
GS5AchainR	CTGCAGCTCGAGGCGGCCGCTTAGAAGGCACTTTGGGGCA
GS5BchainF	GAATTCGGGATCCATATGTTTTCAGGCTATGATCCATGC
GS5BchainR	CTGCAGCTCGAGGCGGCCGCTTCTGCTTGTCGGAAGCTT
OsBAK1-4LRRF	ACTAGTATGGAATTCAACTACGAAGTGGTGGCGCTGA
OsBAK1-4LRRR	CTCGAGGATATCATCTCCAGACTTGACACCACAGATC
OsBAK1-7LRRF	ACTAGTATGGAATTCAACAACGAAGTGCAAGCTCTGATTG
OsBAK1-7LRRR	GCGGCCGCGGATCCATCCTGCTCCCTGTTGGCGTC
OsBAK1-9LRRF	ACTAGTATGGAATTCAACTACGAAGTGGCGGCGCTGATG
OsBAK1-9LRRR	CTCGAGGGATCCGCAACCATGCATAATTGATGAATTGC
OsBAK1-4KDF	GGATCCCATATGAGGCGCAATCAGCAGATCTT
OsBAK1-4KDR	ACTAGTGAATTCCCTCGGTCCAGAGAGCTCCA
OsBAK1-7KDF	GGATCCCATATGCGCCGCAACCGGCAGATCCT
OsBAK1-7KDR	ACTAGTGAATTCTCTGGGGCCGGAGAGCTCGA
OsBAK1-9KDF	CATATGGAATTCTGCAGATGGCGCTTGCCTTT
OsBAK1-9KDR	ACTAGTCTCGAGTCTGGGACCAGATAGCTCAATTGG
